# Estimation of static and dynamic functional connectivity in resting‐state fMRI using zero‐frequency resonator

**DOI:** 10.1002/hbm.26606

**Published:** 2024-06-19

**Authors:** Sukesh Kumar Das, Anil K. Sao, Bharat B. Biswal

**Affiliations:** ^1^ School of Computing and Electrical Engineering Indian Institute of Technology Mandi Mandi Himachal Pradesh India; ^2^ Department of Computer Science and Engineering Indian Institute of Technology Bhilai Bhilai Chhattisgarh India; ^3^ Department of Biomedical Engineering New Jersey Institute of Technology Newark New Jersey USA

**Keywords:** coactivation pattern (CAP), conditional rate map, functional connectivity, resting‐state fMRI, spontaneous BOLD event, zero‐frequency resonator

## Abstract

Resting‐state functional magnetic resonance imaging (rs‐fMRI) is increasingly being used to infer the functional organization of the brain. Blood oxygen level‐dependent (BOLD) features related to spontaneous neuronal activity, are yet to be clearly understood. Prior studies have hypothesized that rs‐fMRI is spontaneous event‐related and these events convey crucial information about the neuronal activity in estimating resting state functional connectivity (FC). Attempts have been made to extract these temporal events using a predetermined threshold. However, the thresholding methods in addition to being very sensitive to noise, may consider redundant events or exclude the low‐valued inflection points. Here, we extract the event‐related temporal onsets from the rs‐fMRI time courses using a zero‐frequency resonator (ZFR). The ZFR reflects the transient behavior of the BOLD events at its output. The conditional rate (CR) of the BOLD events occurring in a time course with respect to a seed time course is used to derive static FC. The temporal activity around the estimated events called high signal‐to‐noise ratio (SNR) segments are also obtained in the rs‐fMRI time course and are then used to compute static and dynamic FCs during rest. Coactivation pattern (CAP) is the dynamic FC obtained using the high SNR segments driven by the ZFR. The static FC demonstrates that the ZFR‐based CR distinguishes the coactivation and non‐coactivation scores well in the distribution. CAP analysis demonstrated the stable and longer dwell time dominant resting state functional networks with high SNR segments driven by the ZFR. Static and dynamic FC analysis underpins that the ZFR‐driven temporal onsets of BOLD events derive reliable and consistent FCs in the resting brain using a subset of the time points.

## Graphical abstract

Blood oxygen level‐dependent (BOLD) events are extracted from the resting‐state fMRI time courses using a zero‐frequency resonator. The BOLD events are used to derive the functional connectivity (FC). Conditional rate map (CRM) and high signal‐to‐noise ratio correlation map are the static FC and coactivation pattern is the dynamic FC.

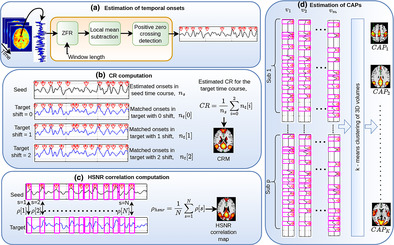

**Practitioner Points**

- Estimation of spontaneous BOLD events and high SNR temporal segments using ZFR from resting‐state fMRI signal.
- Static functional connectivity analysis using estimated BOLD events and high SNR segments.
- Dynamic functional connectivity analysis using estimated high SNR segments.

## 1 INTRODUCTION

Resting‐state functional magnetic resonance imaging (rs‐fMRI) is a popular neuroimaging technique to infer the functional organization of the brain in healthy and different clinical disorders (Rashid et al., 2016). The ease of acquisition has made the rs‐fMRI a popular choice over the task‐based fMRI, especially for populations including infants, stroke, and traumatic brain injury (TBI) who may not be able to satisfactorily perform the cognitive tasks as required in task‐based fMRI (Mwansisya et al., 2017). fMRI measures local changes in deoxyhemoglobin concentration activity through neurovascular coupling (Ogawa et al., 1992). This coupling is modeled as a linear time‐invariant system in a specific brain region (or a voxel) and is called the hemodynamic response function (HRF). It relates the observed signal to the neuronal activity representing a source of delay and a variable physiological process across brain regions (Roebroeck et al., 2011; Valdes‐Sosa et al., 2011). The temporal deconvolution of neuronal activity signal (NAS) or activity‐inducing signal assumes that the blood oxygen level‐dependent (BOLD) time course is an output of the linear HRF with some additive noise. Thus, the observed BOLD fMRI time course ($m\left[n\right]$) can be written as (Boynton et al., 1996):

(1) $$m\left[n\right]=s\left[n\right]*h\left[n\right]+\varepsilon \left[n\right],$$

where n and ${\;}^{*}$ denote the time index and convolution operation respectively. The HRF denoted as $h\left[n\right]$, models the hemodynamic coupling, and $\varepsilon \left[n\right]$ is the noise evoked during measurement. $s\left[n\right]$ is the underlying NAS that carries timing information of neuronal activity. Hypothetically, it is modeled as a train of impulse functions and the temporal onsets of impulses can be used in the deconvolution procedure (Das et al., 2021; Glover, 1999; Wu et al., 2013).

For task fMRI, external stimuli are used as prior information to estimate the NAS from the observed BOLD time course (Bush & Cisler, 2013; Glover, 1999; Sreenivasan et al., 2014; Wink et al., 2008). The external stimuli may introduce some latency but can be considered as a strong neuronal surrogate for the NAS and are used in deconvolution. However, this direct input is not available for rs‐fMRI as no external stimuli are presented at the time of the data acquisition. So, it has no explicit neuronal surrogate while using deconvolution methods (Cherkaoui et al., 2019; Cherkaoui et al., 2021). A few studies have been carried out to deconvolve the NAS from the observed time course in rs‐fMRI (Cherkaoui et al., 2019, 2021; Karahanoğlu et al., 2013; Wu et al., 2013). The deconvolution methods either consider a data‐driven activity surrogate or a model of HRF. The NAS‐surrogates using a threshold (Wu et al., 2013) typically include redundant temporal event points or may exclude some comparatively low‐valued crucial points associated with neuronal excitation. As the neuronal excitation is reflected in the BOLD responses but in a sluggish manner, a threshold includes redundant time points in a high BOLD‐valued temporal region. Some of the approaches do not use a surrogate of NAS and attempt the deconvolution problem with the assumption of an HRF defined by its uniformity (Karahanoğlu et al., 2013) or a limited number of parameters (Cherkaoui et al., 2019, 2021) and may not interpret the true coupling. Thus, the estimate of the NAS may not be accurate and therefore, a precise neuronal surrogate or a robust model of HRF in rs‐fMRI is necessary for deconvolution.

Recent studies have demonstrated that the high‐valued BOLD signal encodes critical information from rs‐fMRI; hence, the resting‐state time course is called a “spontaneous event related” to randomly occurring events (Petridou et al., 2013; Wu et al., 2013). Such events can be traced by estimating the precise temporal onsets of BOLD events. Point process analysis (PPA) is an efficient way to estimate the onsets. The PPA, in rs‐fMRI, has been applied for data reduction by preserving the important information in the BOLD signal. Generally, it is performed by choosing a predefined threshold at the voxel time course by considering only time points above the threshold in the time courses. Using PPA, resting BOLD event‐triggered averages (rBeta) were computed from the high amplitude BOLD fluctuations while the time course exceeded a predefined threshold (Tagliazucchi et al., 2011). The high‐valued BOLD signals are assumed to be the results of neuronal excitation and are used to reduce physiological noise and are also used in deconvolution (Li et al., 2014; Wu et al., 2013; Wu & Marinazzo, 2015). The selected time points, using thresholding, are further extended to explore FCs (conditional rate map—CRM and coactivation pattern—CAP) in the brain (Cifre et al., 2020; Freitas et al., 2020; Liu & Duyn, 2013; Tagliazucchi et al., 2012; Tagliazucchi et al., 2016; Zhuang et al., 2018). An association of variations of functional connectivity (FC) has also been established with the higher and lower intrinsic activity of a network by using a suitable threshold (Di & Biswal, 2015). In most of the above studies, PPA has been employed in resting‐state fMRI and it shows that only a few selected time points, instead of the entire time periods of the BOLD signal, are sufficient to estimate the FC without significant loss of precision.

The BOLD events in the observed rs‐fMRI time course are the consequences of spontaneous neuronal activity. In this manuscript, we hypothesize that the spontaneous events of an rs‐fMRI time course can be estimated using a zero‐frequency resonator (ZFR). We have recently demonstrated the use of ZFR in task fMRI to reliably estimate the instantaneous changes (events) in the fMRI time course (Das et al., 2023). We have also demonstrated in task fMRI (Das et al., 2023) that the ZFR can outperform the threshold‐based PPA discussed earlier. Here, the ZFR is used to estimate the spontaneous BOLD events (temporal) from the rs‐fMRI time course. The estimated events and samples around the events (high signal‐to‐noise ratio [SNR] segments) are used to derive the static and dynamic connectivity and reveal the spontaneous functional organization in the brain. The static FCs are obtained using predefined seeds and computing the conditional rate (CR) or an average of aggregated correlation in high SNR (HSNR) regions. The static FC demonstrates that the ZFR‐based CRs for the coactivations and non‐coactivations (voxels) are well separated. The dynamic FCs obtained using CAP analysis demonstrate that the HSNR driven by the ZFR produced stable functional networks and higher dwell time for dominant resting state networks in comparison with the CAPs obtained from the entire time series.

## 2 MATERIALS AND METHODS

### 2.1 Methods

**FIGURE 1:**
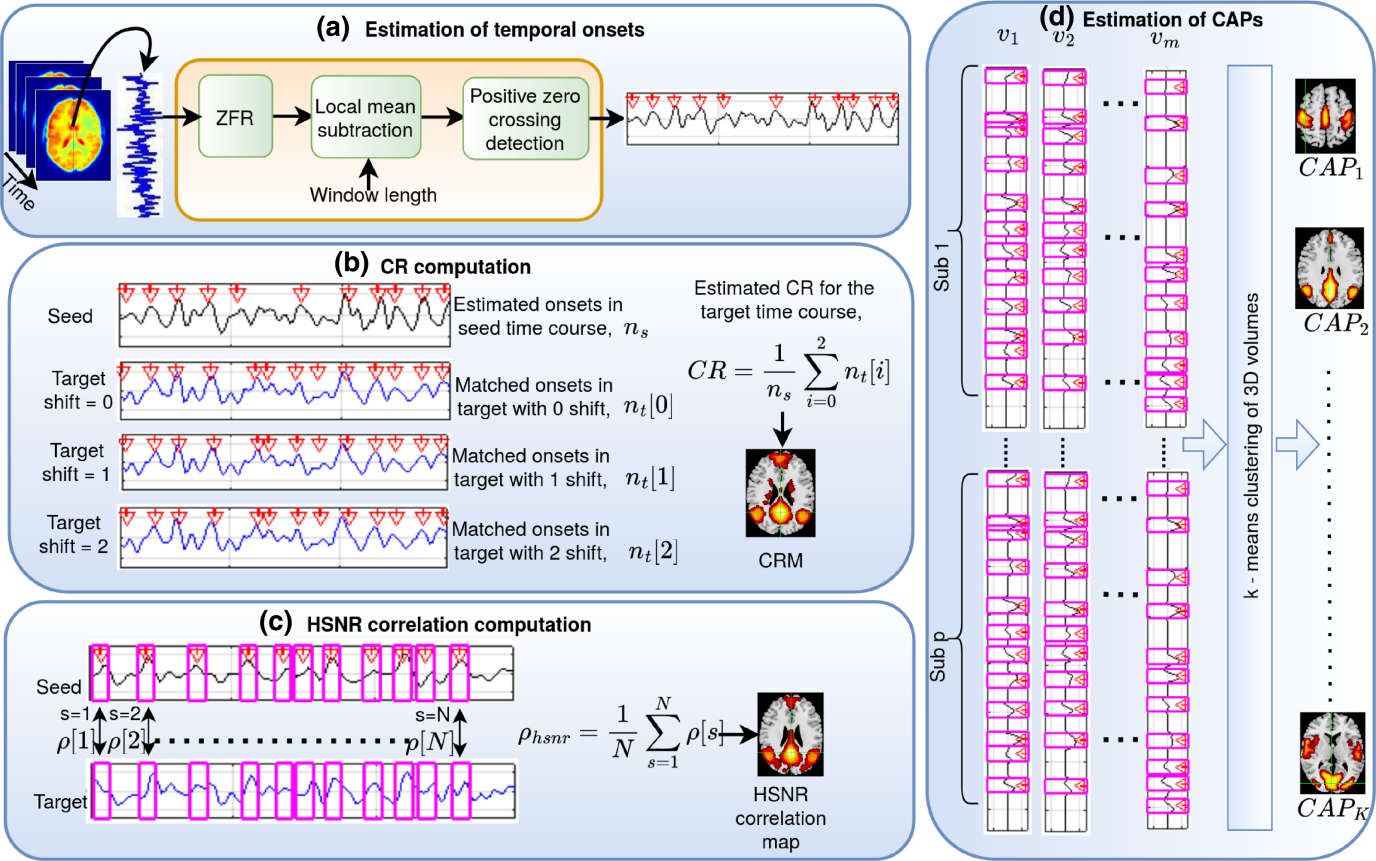
Schematics illustrating the estimation of FC using the onsets of the BOLD event. (a) Extraction of temporal onsets of the BOLD events using the ZFR method. The local mean is subtracted at the output of the ZFR and the positive zero crossing provides the onset locations. (b) CR was computed for every target voxel with respect to the seed voxel. Target onsets are matched with the seed onsets and then the target signal is shifted. Total matched onsets are computed in every shift and the sum of these divided by the number of temporal onsets in the seed time courses yields the CR as given in the equation. CR across all voxels with respect to a seed produces a conditional rate map (CRM—static FC). (c) Samples around the estimated onsets were considered the HSNR segments in the time courses. Correlations between the HSNR segments in the seed time course and the corresponding locations in the target time course are computed. The average of the aggregated correlation for all voxels in the brain produces the HSNR correlation map (static FC). $\rho_{\text{hsnr}}$ is the HSNR correlation and $\rho \left[s\right]$ is the correlation at segment s. (d) At every voxel ($v_{1},v_{2}.\ldots v_{m}$), estimated HSNR segments are considered and appended for all p subjects. Then, all time frames are clustered into K clusters using *k*‐means clustering. Centers of all clusters are considered as CAPs (dynamic FC). Every CAP is then processed through intensity thresholding (top 20% after min–max normalization), spatial clustering (considered the voxels with clusters consisting of at least 16 neighboring voxels). BOLD, blood oxygen level‐dependent; CR, conditional rate; FC, functional connectivity; HSNR, high signal‐to‐noise ratio; ZFR, zero‐frequency resonator.

Estimation of underlying spontaneous neuronal information (delayed) in the form of temporal onsets of the BOLD events using ZFR and computation of static and dynamic FCs have been outlined in the following block diagram (Figure 1). Panel (a) demonstrates how temporal onsets of the BOLD events are estimated using the ZFR method in rs‐fMRI. CR and an average of aggregated HSNR correlation computation to derive static FC, are illustrated in panels (b) and (c), respectively. Panel (d) demonstrates the deriving of CAPs which corresponds to different functional networks in the brain.

The ZFR has been introduced to estimate the onsets of the BOLD events in task‐related fMRI (Das et al., 2023). The current work explores the ZFR to estimate the spontaneous BOLD events in rs‐fMRI and static and dynamic FCs are derived using these estimated events. The narrow‐band filtering of the BOLD signal around zero frequency provides evidence of the temporal onsets of the transient activity in the time course. Thus, the output of ZFR for a resting state BOLD time course will preserve mostly the information of the temporal onsets of the BOLD events. An ideal ZFR is a second‐order system whose system function is given by (Murty et al., 2009; Prasanna & Pradhan, 2011; Yegnanarayana & Murty, 2009)

(2) $$H_{z}\left(Z\right)=\frac{Y\left(z\right)}{M\left(z\right)}=\frac{1}{1-2z^{-1}+z^{-2}},$$

where $Y\left(z\right)$ and $M\left(z\right)$ are the z‐transform of output $y\left[n\right]$ and input $m\left[n\right]$ of a ZFR, respectively. The output of the ZFR for a BOLD time course will grow approximately as a polynomial function of time. However, the alteration in the output contains the temporal onsets of the BOLD events. To extract the location of the events, the local mean is subtracted from the output of the ZFR. The resulting signal is given by

(3) $$z\left[n\right]=y\left[n\right]-\frac{1}{2N_{1}+1}\sum_{k=-N_{1}}^{N_{1}}y\left[n-k\right],$$

where $\left(w_{z}=2N_{1}+1\right)$ is the size of the window in the samples. The positive zero‐crossings in the filtered signal ($z\left[n\right]$), where the signal swings its sign from negative to positive, correspond to the timing of the BOLD events.

#### 2.1.1 Features associated with ZFR output

The ZFR method was used to estimate the temporal onsets of the BOLD events in the time course. The onsets of the events were used to compute CR (Das et al., 2023; Liu et al., 2018; Liu & Duyn, 2013; Tagliazucchi et al., 2012; Wu et al., 2013), a useful metric to estimate static FC. CR between a seed and a target is computed as

(4) $$\mathrm{CR}=\frac{1}{n_{s}}\sum_{i=0}^{2}n_{t}\left[i\right],$$

where $n_{s}$ is the estimated temporal onsets in the seed time course and $n_{t}\left[i\right]$ is the matched onsets in the target time course at *i*th shift with the onsets in the seed time course. If the seed and target voxels are coactivated then the value of the CR will be high, that is, close to 1. On the contrary, if the CR value is low then the seed and target voxels are not coactivated. The delay between the input stimulus and fMRI response can be several seconds. Therefore, in the work, we have included a delay of at most two‐time points for matching the estimated onsets in the target time course with the estimated onsets in the seed time course (Tagliazucchi et al., 2012).

The second feature, HSNR segments, that is, the BOLD values around the estimated events, contains most of the energy ensuring the presence of spontaneous neuronal information in the BOLD time course. Now, this feature is denoted in terms of $l_{o}$ (location of onset). Then the HSNR segment will be estimated as $m\left(l_{o}-1,l_{o}+w-1\right)$, where w indicates the length of the HSNR window. The temporal region, around the onset point, is called the HSNR region because high activity reduces the effect of noise in the measured BOLD time course. The selected HSNR segments of the seed voxel time course and the same temporal regions in target time courses were used to compute Pearson's correlation coefficient and an average of its aggregation is used to quantify the coactivation strength. The HSNR correlation between a seed and a target time course is computed as

(5) $$\rho_{\text{hsnr}}\left(m_{s},m_{t}\right)=\frac{1}{N}\sum_{s=1}^{N}\frac{\sum_{i=l_{0}\left[s\right]-1}^{l_{0}\left[s\right]+w-1}\left(m_{s}\left[i\right]-\mu_{m_{\mathrm{ss}}}\right)\left(m_{t}\left[i\right]-\mu_{m_{\mathrm{ts}}}\right)}{\left(w-1\right)\sigma_{m_{\mathrm{ss}}}\sigma_{m_{\mathrm{ts}}}},$$

where N is the total number of HSNR segments estimated using ZFR, $l_{0}\left[s\right]$ is the location of onset at *s*th HSNR segment, $m_{s}$ is the seed time course, $m_{t}$ is the target time course, $\mu_{m_{\mathrm{ss}}}$ is the mean of the seed time course at *s*th segment, $\mu_{m_{\mathrm{ts}}}$ is the mean of the target time course at *s*th segment, $\sigma_{m_{\mathrm{ss}}}$ is the standard deviation of the seed time course at *s*th segment, and $\sigma_{m_{\mathrm{ts}}}$ is the standard deviation of the target time course at *s*th segment. The rs‐fMRI time courses with only HSNR regions are also used to derive dynamic FC in terms of CAP.

### 2.2 Data description and tools

The experiments for the proposed method were carried out using synthetic and real rs‐fMRI data. The synthetic data were simulated for five subjects using the SimTB toolbox (Erhardt et al., 2012). The data were generated with 150‐time points and TR of 2s. The dimension of the 2D slice was taken as 100×100. Six spatial components were selected and the components are equivalent to default mode network (DMN), dorsal attention network (DAN), primary motor 1 and 2 (M1 and M2), and right and left auditory network (rAud and lAud). Intersubject variability was introduced to the spatial components by spatial variability in translation, rotation, and spread. Four types of events (standard, target, novel, and spike) with probabilities .06, .075, .075, and .05, respectively, are used to convolve with HRF to generate component‐time courses. Voxelwise time courses were then generated as a linear combination of spatial components weighted by the component‐time courses with added baseline tissue weights. Finally, Rician noise was added to the simulated data with random contrast‐to‐noise ratios ranging from 0.2 to 0.65 for different subjects. The given additional description of the synthetic data generation has been provided in the supplementary material (section 5).

The rs‐fMRI data were taken from the UCLA Consortium for Neuropsychiatric Phenomics data, a publicly available data set. A total of 40 healthy participants (age = 21–50, 18F, 22M) are considered in this study. The data were acquired on a 3 T Siemens Trio scanner. The fMRI data were acquired using the following scan parameters: TR (repetition time) = 2000 ms; TE (echo time) = 29 ms; field of view = 192 mm; flip angle = 72°; # slices (axial) = 34; slice thickness = 4 mm with gap = 0; matrix size = 64×64; #images = 152. T1‐weighted images: TR = 1.9 s; TE = 2.26 ms; field of view = 250 mm; # slices = 176; slice thickness = 1 mm. The data were preprocessed using SPM12^1^ with MATLAB R2019a. The initial three‐time points across subjects were discarded to get a steady T1 magnetization. Anatomical images were reoriented with respect to the Montreal Neurological Institute (MNI) space and functional images were reoriented with the anatomical images. Functional images were realigned, followed by slice time correction. Anatomical images were co‐registered with functional images and it was segmented into gray matter, white matter, and cerebrospinal fluid. Functional images were then normalized to MNI space using the deformation field obtained in segmentation. The normalized functional images were smoothed with a Gaussian kernel of FWHM 5mm×5mm×5mm. The time courses are filtered using a band‐pass filter with a range of (0.01−0.1Hz) to reduce the high‐frequency physiological noise.

### 2.3 Data set and code availability

UCLA data are made publicly available (https://openneuro.org/datasets/ds000030/versions/00016). The HCP rest data are also publicly available (https://db.humanconnectome.org/app/action/DownloadPackagesAction). The code of the ZFR for estimating the spontaneous BOLD‐events is available at https://github.com/sentudas32/ZFR_rest.

## 3 RESULTS

The results demonstrate the CR computation to derive the static FC (CRM) and estimation of HNSR segments for static and dynamic FC (CAP). For all the experiments, window lengths ($w_{z}$) of nine and six samples (TR) were considered for ZFR local mean subtraction and the HSNR segments, respectively.

### 3.1 CR computation and HSNR segment estimation

**FIGURE 2:**
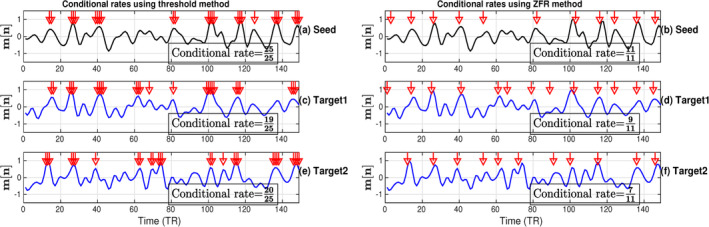
Conditional rate using: onsets estimated by (a) thresholding approach and (b) ZFR approach at a seed voxel time course taken from posterior cingulate cortex (PCC; MNI coordinate: −0, −52, 26), onsets estimated by (c) thresholding approach and (d) ZFR approach at coactive target voxel time courses taken from coactivated right lateral parietal cortex (rLPC; MNI coordinate: 42, −62, 32) and onsets estimated by (e) thresholding approach and (f) ZFR approach at non‐coactive target voxel time courses taken from right auditory cortex (rAud; MNI coordinate: 58, −22, 12), respectively. The ratio of the coactivation to non‐coactivation scores is 0.95 and 1.29 using threshold and ZFR, respectively. MNI, Montreal Neurological Institute; ZFR, zero‐frequency resonator.

**FIGURE 3:**
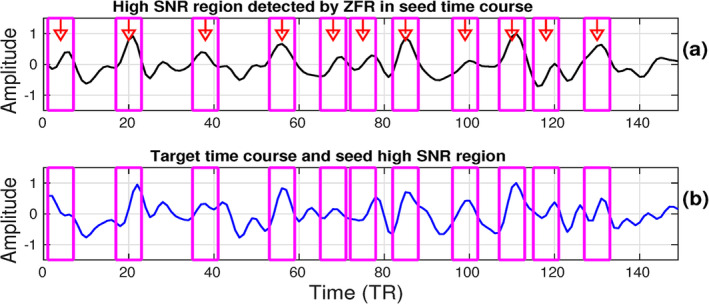
High SNR segments: (a) estimated high SNR in seed time course taken from right parietal cortex—rLPC (MNI coordinate: 42, −62, 32), (b) superimpose seed high SNR region on target time course taken from left parietal lobe (MNI coordinate: −48, −62, 34). MNI, Montreal Neurological Institute; SNR, signal‐to‐noise ratio; ZFR, zero‐frequency resonator.

CR computation for two target voxels (one coactivated and the other non‐coactivated) against a chosen seed voxel has been illustrated in Figure 2. The seed voxel is chosen from the posterior cingulate cortex (PCC; MNI coordinate: −0, 52, 26) which is in the task‐negative DMN and shown in Figure 2a,b. The coactive (Figures 2c,d) and non‐coactive (Figure 2e,f) target voxels were chosen from the right lateral parietal cortex (rLPC; MNI coordinate: 42, −62, 32) of DMN and right auditory cortex (rAud; MNI coordinate: 58, −22, 12) of auditory task network respectively. The CRs, obtained for the target voxels, as explained in Section 2.1.1 using onsets estimated by thresholding (Figure 2c) and ZFR (Figure 2d), are also shown in the figure respectively. Using the ZFR, the estimated CR for the coactivation was greater than that obtained using the threshold‐based approach. In Figure 2c,d, it can be observed that the CRs are 0.76 and 0.82 for coactivation using the thresholding and the ZFR, respectively. On the other hand, for non‐coactivation, the CRs are 0.80 and 0.63 using the thresholding and the ZFR, respectively (Figure 2e,f). The ratio of the coactivation to non‐coactivation scores is 0.95 and 1.29 using threshold and ZFR, respectively. Therefore, the CR using the ZFR‐based approach distinguishes the coactivation and non‐coactivation more fairly than the threshold‐based approach. The high SNR region, proposed as a feature, contains most of the energy ensuring the presence of neuronal information (temporal) in the rs‐fMRI time course. Extracted HSNR regions in the observed time course were demonstrated for a seed voxel time course in Figure 3a and the same temporal regions were overlaid on the target time course (Figure 3b).

### 3.2 Static FC analysis on synthetic data

**FIGURE 4:**
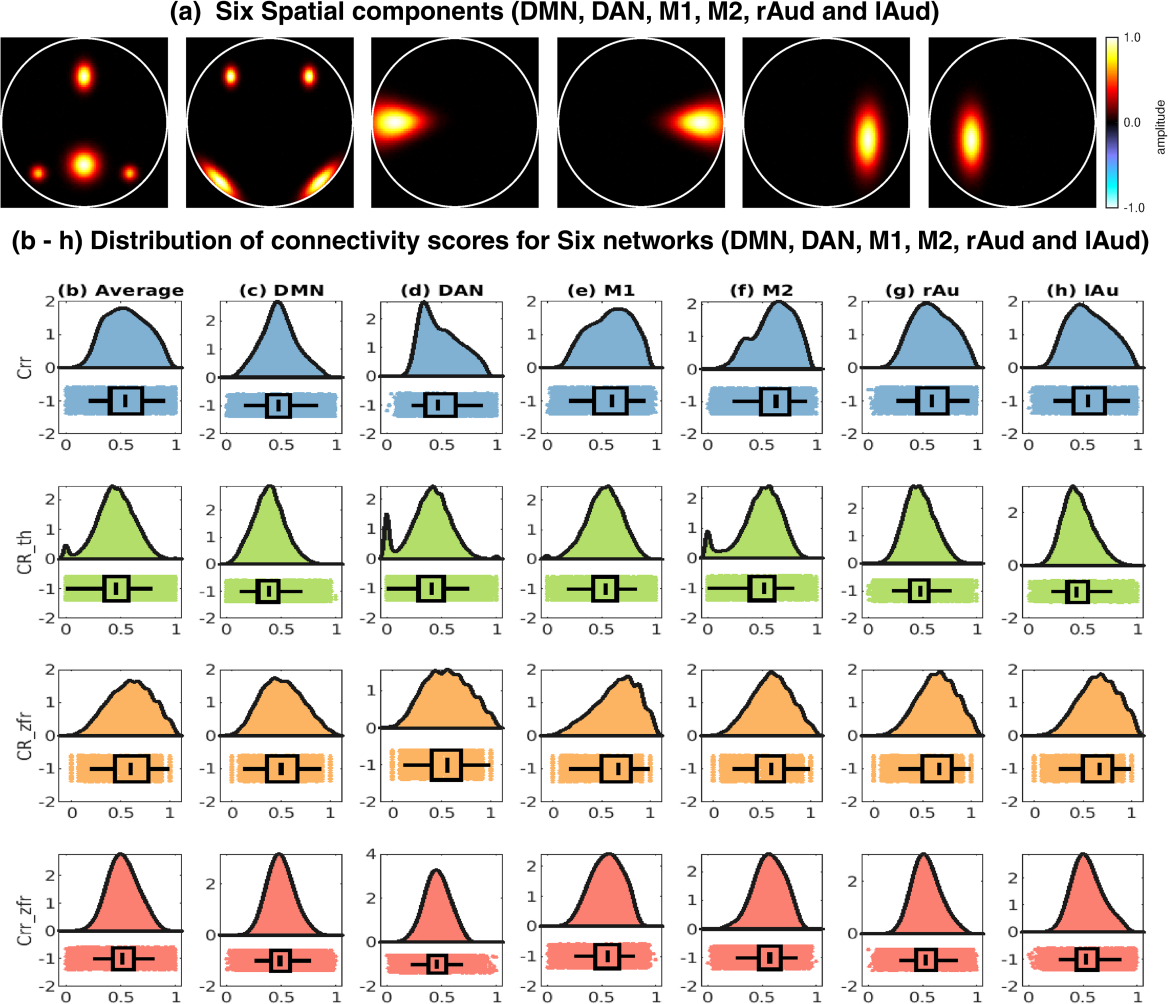
Six synthetically generated spatial maps and behavior of the connectivity scores obtained for different spatial components using four metrics (correlation, CR with thresholding, CR with ZFR, and HSNR correlation with ZFR) in the corresponding networks. The connectivity scores are obtained with the time courses from the active area of the respective spatial maps and from five subjects. Here, Crr, CR_th, CR_zfr, and Crr_zfr stand for correlation, CRs with thresholding method, CRs with ZFR method, and HSNR correlation using ZFR method, respectively. (a) Six synthetically generated spatial maps corresponding to six networks which can be considered as the ground truth of the six different networks equivalent to default mode network (DMN), dorsal attention network (DAN), motor 1 (M1), motor 2 (M2), right auditory (rAud) and left auditory (lAud) of the resting state networks. Distributions for (b) all six networks, (c) DMN, (d) DAN, (e) M1, (f) M2, (g) for rAud, and (h) lAud are obtained using the correlation, threshold‐based CR, ZFR‐based CR, and ZFR‐based HSNR correlation respectively (from top to bottom). CR, conditional rate; HSNR, high signal‐to‐noise ratio; ZFR, zero‐frequency resonator.

Six spatial components were considered to simulate the five subjects' data. The ground truth of the spatial maps is demonstrated in Figure 4a. Active voxels (the voxels lying on the activated regions in the ground truth spatial component or network. $n_{a}$ in number) are obtained for every component from the corresponding ground truth spatial components and connectivity scores (degree of similarity computed using any metric) were computed between the average time course of the voxels lying in the active area and other voxels in the area using the four metrics (correlation, CR using threshold, CR using the ZFR, and the ZFR‐based HSNR correlation). A total of $p\times n_{a}$ coactivation scores are obtained for every component and p subjects using one of the four metrics. The distributions of the scores for the 6 components (or networks) are shown in Figure 4c–h. The average distribution of all networks is shown in Figure 4b. It can be noticed that overall variance is less in the case of the ZFR‐based HSNR correlation. The ZFR‐based CR shows a higher mean in connectivity scores inside the networks in comparison to other matrices and smaller variances in comparison to the threshold‐based CR. A pair *t* test has been performed between the metrics (correlation, threshold‐based CR, ZFR‐based CR, and ZFR‐based correlation). For each case, it rejects the null hypothesis ($H_{0}$) resulting in the variances being different (p<.05).

### 3.3 Static FC analysis on real data

**FIGURE 5:**
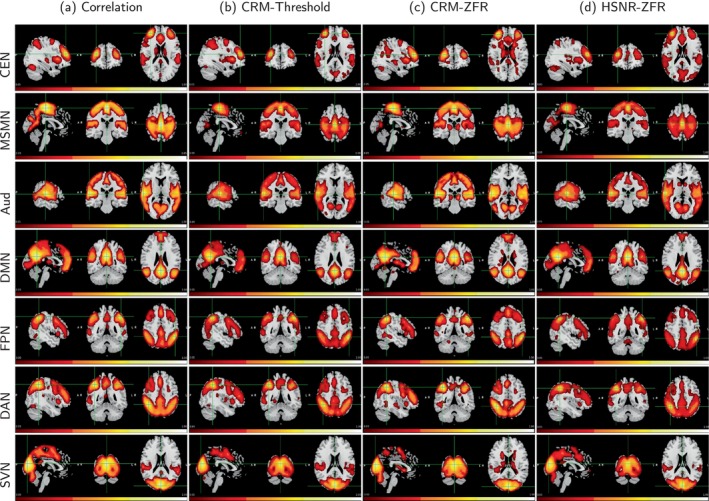
Sagittal, coronal, and axial views of 40 subjects' static FC (UCLA) with respect to seven seeds (from top: CEN—MNI coordinate 34, 46, 20; MSMN—MNI coordinate 0, −24, 60; Aud—MNI coordinate 58, −24, 11; DMN—MNI coordinate 0, −52, 26; FPN—MNI coordinate −48, −47, 49; DAN—MNI coordinate 40, −47, 48; and SVN—MNI coordinate 0, −85, 20). (a) Correlation map using entire time courses, (b) and (c) CRMs using the estimated onsets by threshold and ZFR, respectively, and (d) Connectivity map using an average of aggregated correlation in HSNR segments estimated by ZFR. All connectivity maps are presented using the same scale (0.05–1). Aud, auditory network; CEN, central executive network; FPN, frontoparietal network; HSNR, high signal‐to‐noise ratio; MNI, Montreal Neurological Institute; MSMN, medial sensory‐motor network; SVN, superior visual network; ZFR, zero‐frequency resonator.

**FIGURE 6:**
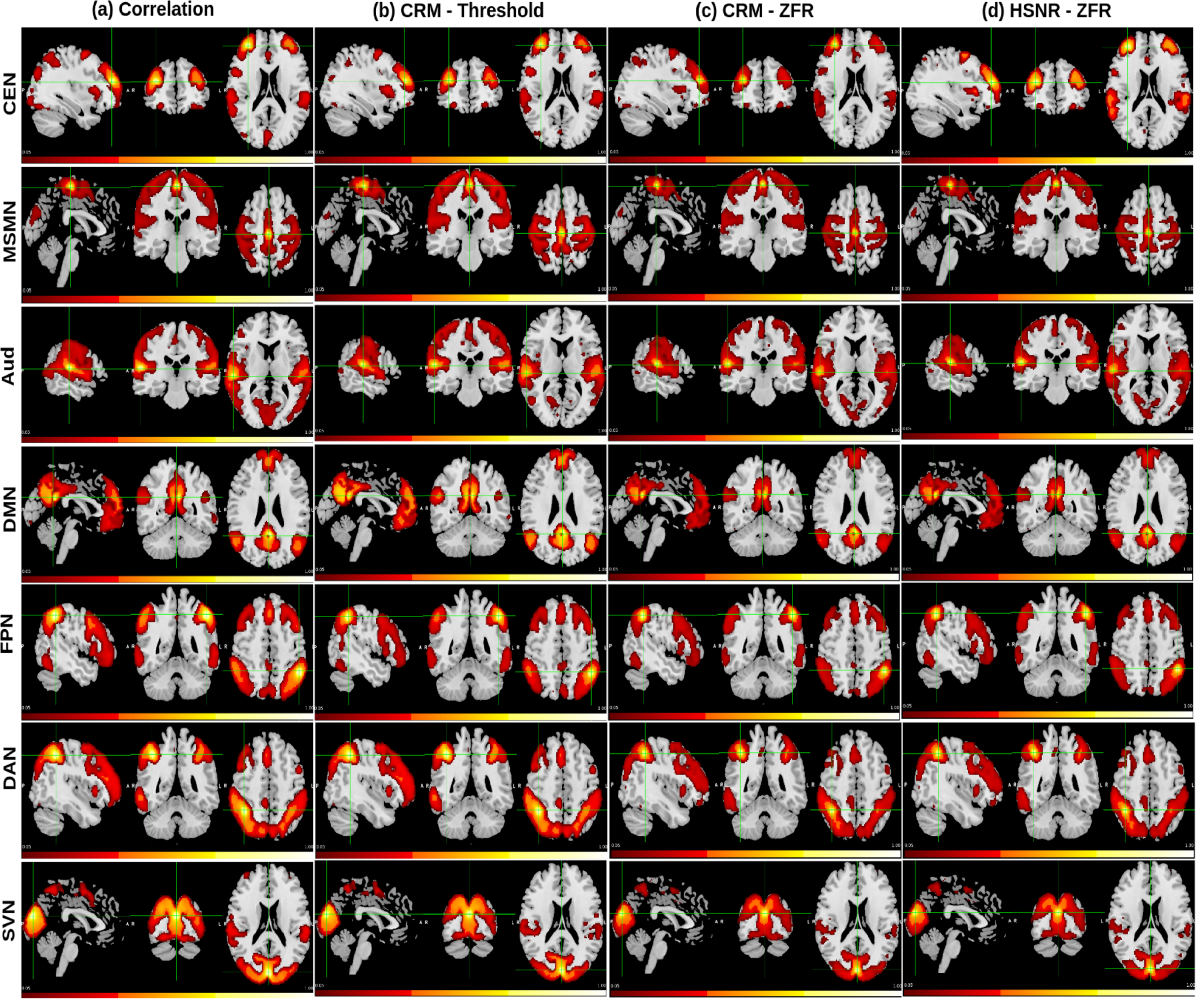
Sagittal, coronal, and axial views of 50 subjects' static FC (HCP data) with respect to seven seeds (from top: CEN—MNI coordinate 34, 46, 20; MSMN—MNI coordinate 0, −24, 60; Aud—MNI coordinate 58, −24, 11; DMN—MNI coordinate 0, −52, 26; FPN—MNI coordinate −48, −47, 49; DAN—MNI coordinate 40, −47, 48; and SVN—MNI coordinate 0, −85, 20). (a) Correlation map using entire time courses, (b) and (c) CRMs using the estimated onsets by thresholding and ZFR, respectively, and (d) Connectivity map using an average of aggregated correlation in HSNR segments estimated by ZFR. All connectivity maps are presented using the same scale (0.05–1). Aud, auditory network; CEN, central executive network; CRM, conditional rate map; FPN, frontoparietal network; HSNR, high signal‐to‐noise ratio; MNI, Montreal Neurological Institute; MSMN, medial sensory‐motor network; SVN, superior visual network; ZFR, zero‐frequency resonator.

It has been shown that a fraction of time points can explain the FC pattern and the CR using a few time points can be used to estimate the static functional network‐like correlation‐based connectivity computed by considering the entire time course data (Tagliazucchi et al., 2012). Seven resting‐state networks (RSNs) named central executive network (CEN), medial sensory‐motor network (MSMN), auditory network (Aud), DMN, frontoparietal network (FPN), DAN, and superior visual network (SVN) have been illustrated in Figures 5 and 6 for UCLA and HCP, respectively. The MNI coordinates of the seed locations for the RSNs are (34, 46, 20), (0, −24, 60), (58, −24, 11), (0, −52, 26), (−48, −47, 49), (40, −47, 48), and (0, −85, 20), respectively. The voxel‐wise correlations were computed with respect to the seeds and the processes were executed for all 40 subjects. The average of the 40 subjects' connectivity maps (UCLA) is shown in Figure 5a. The voxel‐wise temporal onsets of the BOLD events were estimated by the threshold and ZFR‐based approaches followed by CR computation as explained in Section 2.1.1 and the CRM for every subject was obtained. The average CRMs of all the subjects are shown in Figure 5b,c using threshold and ZFR‐based methods, respectively. Furthermore, FC was computed using HSNR segments across the voxels in the brain with respect to the seed as explained in Equation (5). Finally, the average spatial map across all the subjects leads to an HSNR correlation map (shown in Figure 5d). For each of the connectivity maps, the top 20% of the voxels were considered after performing min‐max normalization. After thresholding, we accounted for clusters of voxels that were connected with at least 16 active neighborhood voxels. It can be observed that the ZFR‐driven CRM and HSNR correlation map (Figure 5c,d) can produce connectivity using less number of plausible time points. A similar observation has also been made for HCP data in Figure 6.

**FIGURE 7:**
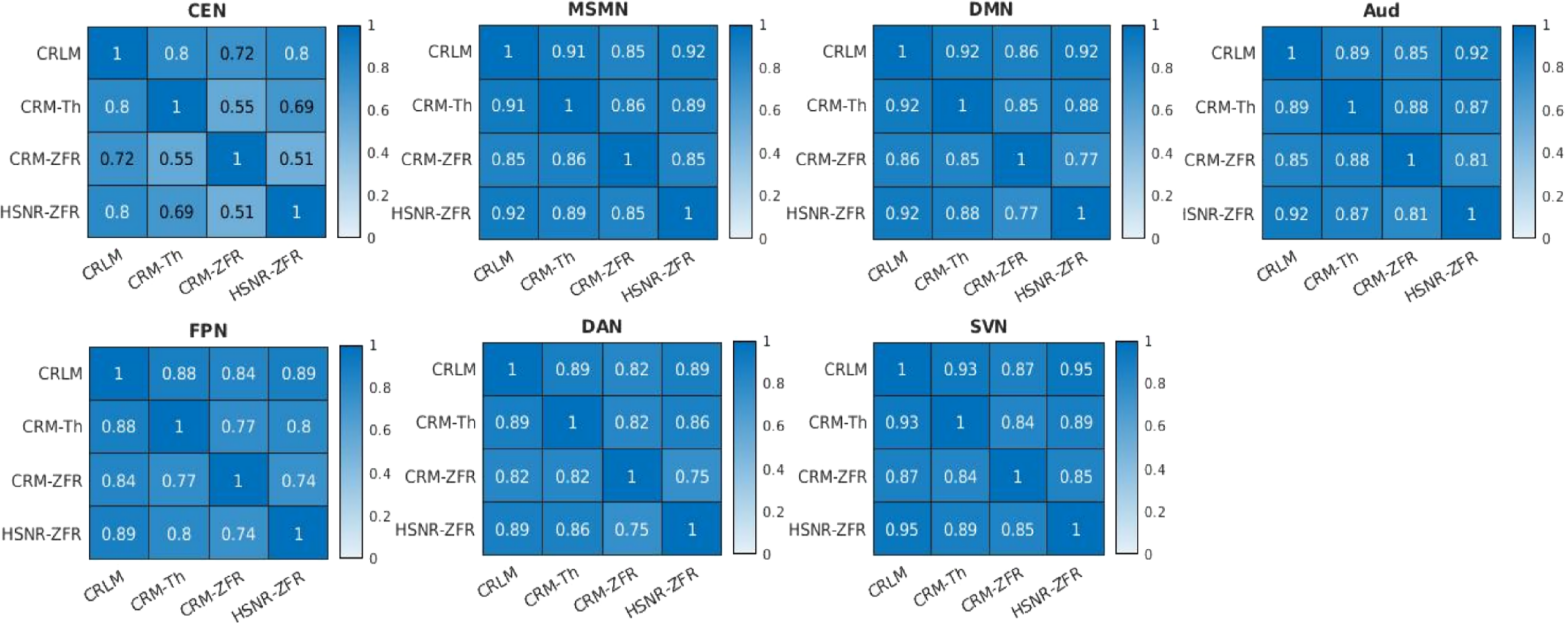
Spatial correlation between the static FCs (CEN, MSMN, DMN, Aud, FPN, DAN, and SVN) derived by the correlation, threshold‐based CR, ZFR‐based‐CR and ZFR‐based HSNR correlation. Here, CRLM, CRM‐Th, CRM‐ZFR, and HSNR‐ZFR stand for the FC maps using correlation, threshold‐based conditional rate, ZFR‐based conditional rate, and ZFR‐based HSNR correlation. AUd, auditory network; CEN, central executive network; DAN, dorsal attention network; DMN, default mode network; FC, functional connectivity; FPN, frontoparietal network; HSNR, high signal‐to‐noise ratio; MSMN, medial sensory‐motor network; SVN, superior visual network; ZFR, zero‐frequency resonator.

The spatial correlations between the maps obtained using different metrics (correlation, threshold‐based CR, ZFR‐based CR, and ZFR‐based HSNR correlation) have been illustrated in the Figure 7. It can be noticed that the spatial correlation is higher between the maps obtained by the correlation with the entire time course and the maps obtained using ZFR‐based HSNR correlation. To analyze the FC scores for coactivation and non‐coactivation, we have considered seven functional networks (default mode—DMN, Somato motor dorsal—SMD, Auditory—Aud, Visual—Vis, Fronto parietal—FPN, Salience—Sal, and Dorsal attention—DAN). They consist of 65, 42, 12, 36, 34, 13, and 16 ROIs respectively from the Seitzman300 atlas (Seitzman et al., 2020) and average time courses from ROIs were used to compute connectivity scores. The correlation and CR scores (based on thresholding and ZFR) were computed for every ROI time course across the brain with respect to the other ROI time courses. The score between two ROIs from the same functional network is called a coactivation score and if the ROIs are from different networks, it is called a non‐coactivation score. Details regarding the coactivation and non‐coactivation scores for seven networks can be observed in Table 1. For the table, a paired *t* test has been performed between two connectivity scores (coactivation and non‐coactivation) for each of the seven networks using the metrics (correlation, threshold‐based CR, ZFR‐based CR, and ZFR‐based correlation). For each case, it rejects the null hypothesis ($H_{0}$) resulting the mean of the connectivity scores of coactivation and non‐coactivation is different (p<.05). The actual p values are .0042, .0046, .00067, and .000086 for correlation, threshold‐based CR, ZFR‐based CR, and HSNR ZFR, respectively, for all networks. It can be noticed that the difference in the mean is more for ZFR‐based CR than that for threshold‐based CR and the same can also be noticed higher for the ZFR‐based HSNR correlation than that for the correlation using entire time points. The variation in coactivation scores is comparatively less in the case of the HSNR‐based correlation. We have also computed the Fisher discriminant ratio (J) for all seven networks using the four types of metric as $J=\frac{{\left|\mu_{a}-\mu_{n}\right|}^{2}}{\sigma_{a}^{2}+\sigma_{n}^{2}}$, where $\mu_{a}$ and $\mu_{n}$ are the mean of the coactivation and non‐coactivation scores, respectively. The $\sigma_{a}^{2}$ and $\sigma_{n}^{2}$ are the variance of the coactivation and non‐coactivation scores, respectively. The obtained J for the seven networks (DMN, SMD, Aud, Vis, FPN, Sal, and DAN) are (0.010, 0.017, 0.039, and 0.064), (0.020, 0.066, 0.051, and 0.020), (0.102, 0.236, 0.192, and 0.142), (0.010, 0.248, 0.232, and 0.320), (0.009, 0.017, 0.030, and 0.054), (0.035, 0.101, 0.103, and 0.080), and (0.033, 0.062, 0.066, and 0.086) respective using correlation, threshold‐based CR, ZFR‐based CR and ZFR‐based HSNR correlation respectively. It can be observed that the ZFR‐based metrics (CR and HSNR correlation) produce significant Fisher discriminant ratio to distinguish between coactivation and non‐coactivation scores.

**TABLE 1:** Mean coactivation and non‐coactivation scores using four different metrics.

Functional network	Metric	Coactivation score	Non‐coactivation score	Difference
DMN	Correlation	0.66±0.14	0.64±0.14	0.02
	CR (threshold)	0.37±0.16	0.34±0.16	0.03
	CR (ZFR)	0.44±0.22	0.38±0.21	0.04
	Correlation (high SNR‐ZFR)	0.65±0.14	0.60±0.14	0.05
SMD	Correlation	0.69±0.16	0.66±0.14	0.03
	CR (threshold)	0.43±0.17	0.37±0.16	0.06
	CR (ZFR)	0.48±0.23	0.41±0.21	0.07
	Correlation (high SNR‐ZFR)	0.66±0.16	0.63±0.14	0.03
Aud	Correlation	0.71±0.16	0.64±0.15	0.07
	CR (threshold)	0.50±0.16	0.39±0.16	0.11
	CR (ZFR)	0.56±0.21	0.43±0.21	0.13
	Correlation (high SNR‐ZFR)	0.70±0.15	0.62±0.15	0.08
Vis	Correlation	0.66±0.14	0.64±0.15	0.02
	CR (threshold)	0.49±0.18	0.37±0.16	0.12
	CR (ZFR)	0.55±0.23	0.40±0.21	0.15
	Correlation (high SNR‐ZFR)	0.73±0.15	0.61±0.15	0.12
FPN	Correlation	0.65±0.15	0.63±0.14	0.02
	CR (threshold)	0.38±0.16	0.35±0.16	0.03
	CR (ZFR)	0.43±0.21	0.38±0.20	0.05
	Correlation (high SNR‐ZFR)	0.66±0.14	0.61±0.15	0.05
Sal	Correlation	0.69±0.16	0.65±0.14	0.04
	CR (threshold)	0.44±0.16	0.37±0.15	0.07
	CR (ZFR)	0.49±0.22	0.39±0.22	0.10
	Correlation (high SNR‐ZFR)	0.68±0.15	0.62±0.15	0.06
DAN	Correlation	0.70±0.16	0.66±0.15	0.04
	CR (threshold)	0.44±0.17	0.38±0.17	0.06
	CR (ZFR)	0.50±0.22	0.42±0.22	0.08
	Correlation (high SNR‐ZFR)	0.68±0.14	0.62±0.15	0.06

Abbreviations: AUd, auditory network; CR, conditional rate; DAN, dorsal attention network; DMN, default mode network; FPN, frontoparietal network; Sal, Salience; SNR, signal‐to‐noise ratio; Vis, Visual; ZFR, zero‐frequency resonator.

### 3.4 Dynamic FC (CAP) analysis on real data

**FIGURE 8:**
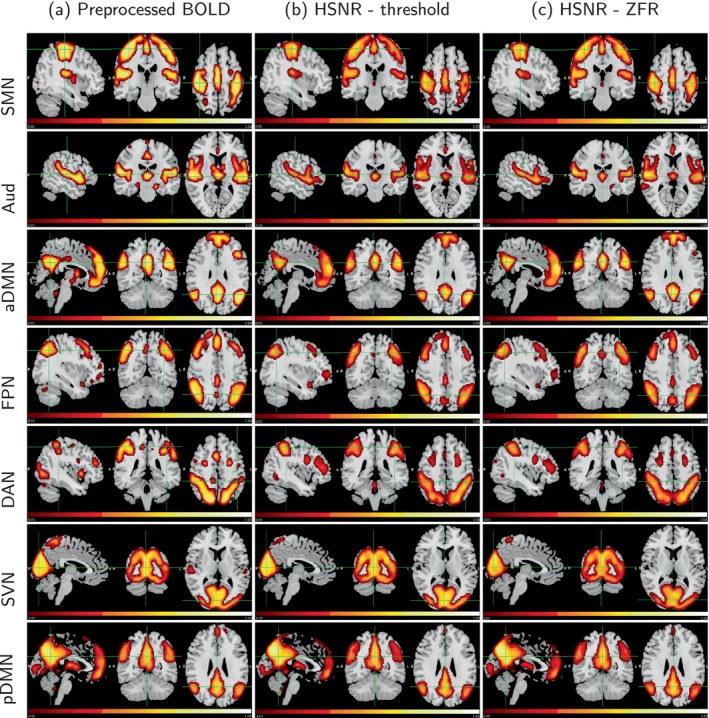
Sagittal, coronal, and axial views of the CAPs (UCLA data set): Seven rows correspond to seven CAPs (from top SMN, Aud, aDMN, FPN, DAN, Vis, and pDMN). CAPs are obtained using (a) preprocessed data, (b) HSNR segments around the onsets estimated by the thresholding, and (c) HSNR segments around the onsets estimated by the ZFR in the rs‐fMRI time course. aDMN, anterior DMN; AUd, auditory network; CAP, coactivation pattern; DAN, dorsal attention network; DMN, default mode network; FPN, frontoparietal network; HSNR, high signal‐to‐noise ratio; pDMN, posterior DMN; rs‐fMRI, resting‐state functional magnetic resonance imaging; SMN, sensory‐motor network; ZFR, zero‐frequency resonator.

**FIGURE 9:**
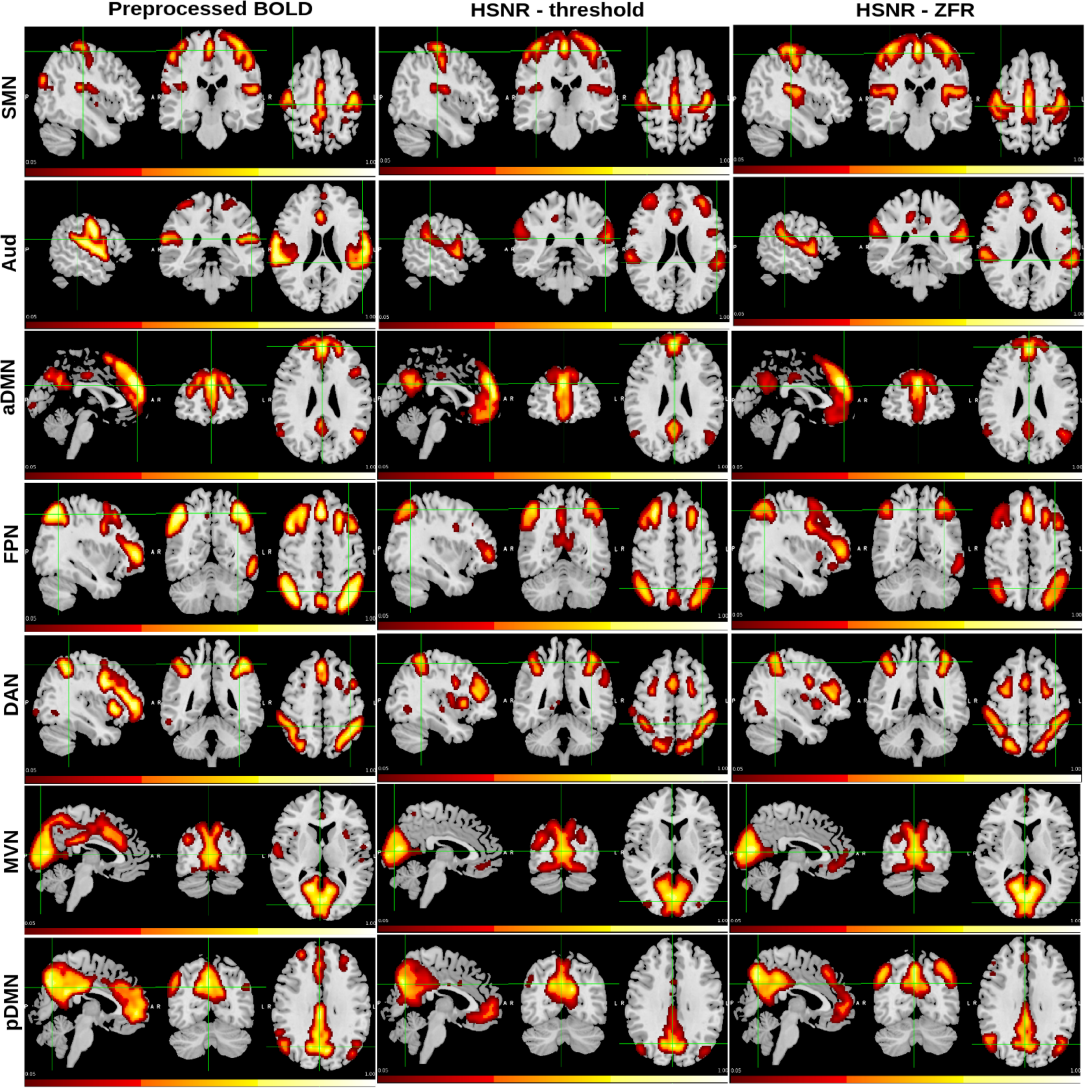
Sagittal, coronal, and axial views of the CAPs (HCP data set): Seven rows correspond to seven CAPs (from top SMN, Aud, aDMN, FPN, DAN, SVN, and pDMN). CAPs are obtained using (a) preprocessed data, (b) HSNR segments around the onsets estimated by the thresholding, and (c) HSNR segments around the onsets estimated by the ZFR in the rs‐fMRI time course. aDMN, anterior DMN; AUd, auditory network; CAP, coactivation pattern; DAN, dorsal attention network; DMN, default mode network; FPN, frontoparietal network; HSNR, high signal‐to‐noise ratio; pDMN, posterior DMN; rs‐fMRI, resting‐state functional magnetic resonance imaging; SMN, sensory‐motor network; ZFR, zero‐frequency resonator.

To investigate the time‐varying information of the resting state data, CAPs were estimated (for both the data set—UCLA and HCP) using three representations of each voxel time course, namely: BOLD values of entire time courses, BOLD values of only HSNRs region around temporal onsets of the BOLD events obtained by thresholding and ZFR approach respectively. The CAPs indicate the specific brain regions, which are similarly active over time (Bray et al., 2015; Chen et al., 2015; Liu et al., 2013; Liu et al., 2018; Liu & Duyn, 2013; Preti et al., 2017). It is estimated by considering fMRI volumes as the fundamental constituent and decomposing it by clustering the time frames using K‐means algorithm with cosine distance as a similarity criterion (Karahanoğlu & Van De Ville, 2015). The center of the time frames of different clusters corresponds to different CAPs, respectively. In this study, K=28 clusters were used in K‐means clustering. This was obtained by the elbow method where the distortions were computed for different K (=15, 16, …, 29, 30) and the value of K $\left(=28\right)$ at the lowest distortion, was considered as the optimal number of clusters. Every CAP image was processed (intensity thresholding and spatial clustering) as explained in Section 3.3. CAPs with three different representations have been demonstrated in Figure 8a–c and in Figure 9a–c. A total of seven CAPs were identified and demonstrated in the figure. The identified CAPs are sensory‐motor network (SMN), Aud, anterior DMN (aDMN), FPN, DAN, SVN, and posterior DMN (pDMN).

**FIGURE 10:**
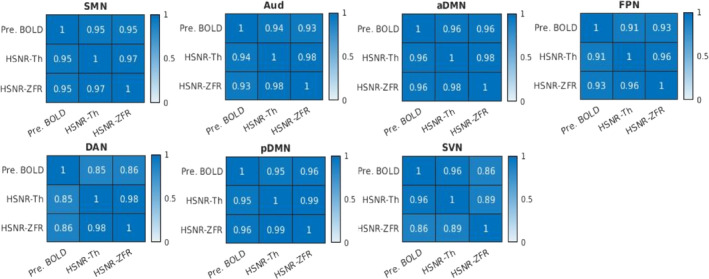
Spatial correlation between the CAPs (SMN, Aud, aDMN, FPN, DAN, pDMN, and SVN) derived by the correlation, threshold‐based HSNR, and ZFR‐based HSNR segments (temporal). Here, Pre‐BOLD, HSNR‐Th, and HSNR‐ZFR stand for the CAPs using preprocessed BOLD time course, threshold‐based HSNR segments, and ZFR‐based HSNR segments. aDMN, anterior DMN; AUd, auditory network; BOLD, blood oxygen level‐dependent; CAP, coactivation pattern; DAN, dorsal attention network; DMN, default mode network; FPN, frontoparietal network; HSNR, high signal‐to‐noise ratio; pDMN, posterior DMN; rs‐fMRI, resting‐state functional magnetic resonance imaging; SMN, sensory‐motor network; ZFR, zero‐frequency resonator.

**FIGURE 11:**
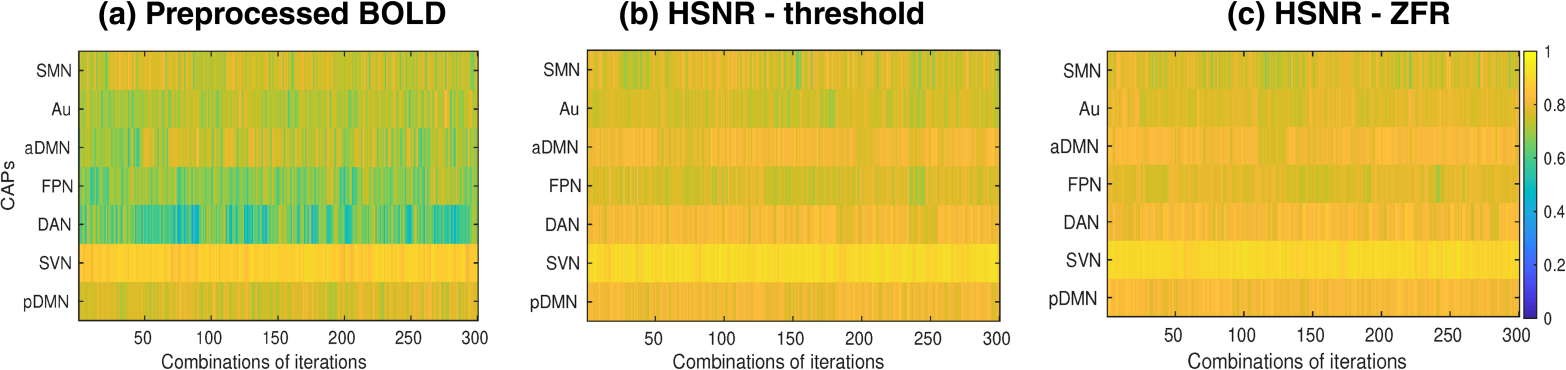
Jaccard similarity distances between CAPs obtained from different random samples. A total of 25 iterations and randomly 70% of the total samples were considered for deriving CAPs in every iteration. A total of 300 similarity values have been demonstrated for every CAP. CAPs are derived using (a) preprocessed BOLD signal, (b) HSNR segments using the threshold method, and (c) HSNR segments using the ZFR‐based method. CAP, coactivation pattern; BOLD, blood oxygen level‐dependent; HSNR, high signal‐to‐noise ratio; ZFR, zero‐frequency resonator.

The spatial correlations between the caps obtained using three representations (preprocessed time course, threshold‐based HSNR segments, and ZFR‐based HSNR segments) have been demonstrated in Figure 10. The spatial correlation between the CAPs using the entire time course and CAPs using the ZFR‐based HSNR segments (smaller number of time points) is also significant. The stability of the CAPs obtained using the three different representations has been demonstrated in Figure 11. Out of a total of 5960‐time frames (40 subjects × 149 time frames), random samples (70% of the total frames) were taken into account for clustering to produce the respective CAPs and it has been repeated for $n_{\mathrm{it}}=25$ times (iterations). In each iteration, the seven CAPs were identified for the three different representations. A Jaccard similarity distance was also computed between the CAPs obtained in different iterations. A total of $\frac{n_{\mathrm{it}}\times \left(n_{\mathrm{it}}-1\right)}{2}=300$ similarity distances for different combinations of CAPs obtained in different iterations using the three representations, have been illustrated in Figure 11a–c. It can be observed that almost all CAPs are comparatively more stable for ZFR‐based HSNR representation. Among all CAPs, SVN gains the highest stability throughout all representations. DAN and FPN are more unstable while using preprocessed BOLD and SMN shows the highest variability using both threshold and ZFR‐based HSNR representations. The average similarities of all CAPs are 0.73±0.089, 0.80±0.053, and 0.81±0.046 using the three representations (preprocessed BOLD, threshold, and ZFR‐based HSNR, respectively).

**FIGURE 12:**
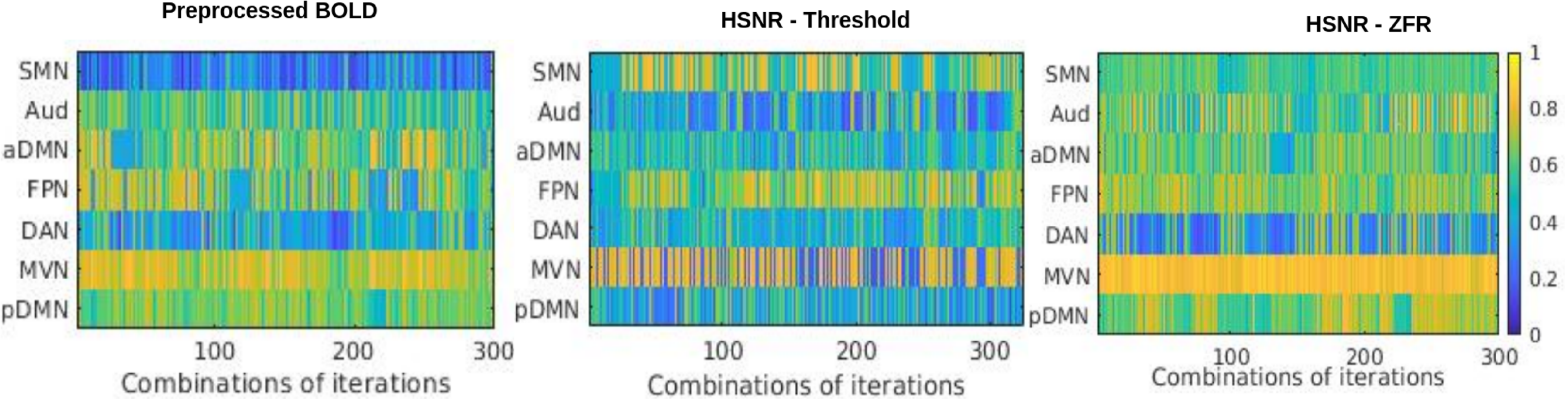
Jaccard similarity distances between CAPs obtained from different random samples (HCP data set). A total of 25 iterations and randomly 70% of the total samples were considered for deriving CAPs in every iteration. A total of 300 similarity values have been demonstrated for every CAP. CAPs are derived using (a) preprocessed BOLD signal, (b) HSNR segments using the threshold method, and (c) HSNR segments using the ZFR‐based method. BOLD, blood oxygen level‐dependent; CAP, coactivation pattern; HSNR, high signal‐to‐noise ratio; ZFR, zero‐frequency resonator.

**FIGURE 13:**
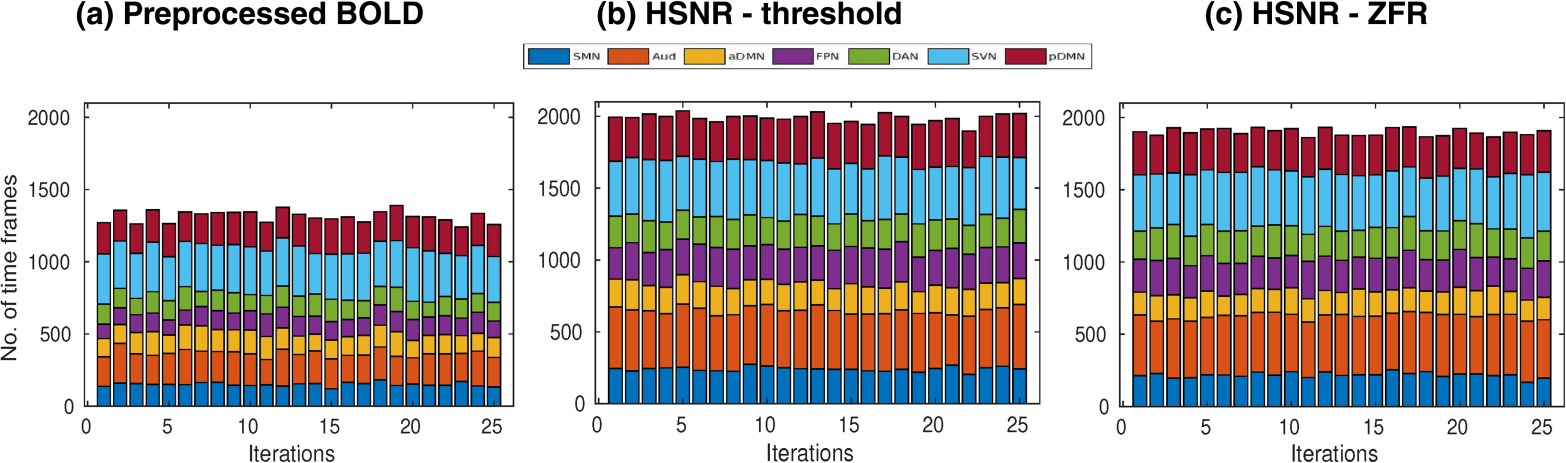
Dwell time (in terms of time frames) of different CAPs obtained from different random samples (UCLA data set) in 25 iterations. Each iteration accounts randomly 70% of the total samples for deriving CAPs. Dwell time has been demonstrated for every CAP in different iterations using (a) preprocessed BOLD signal, (b) HSNR segments using the threshold method, and (c) HSNR segments using the ZFR‐based method. BOLD, blood oxygen level‐dependent; CAP, coactivation pattern; HSNR, high signal‐to‐noise ratio.

**FIGURE 14:**
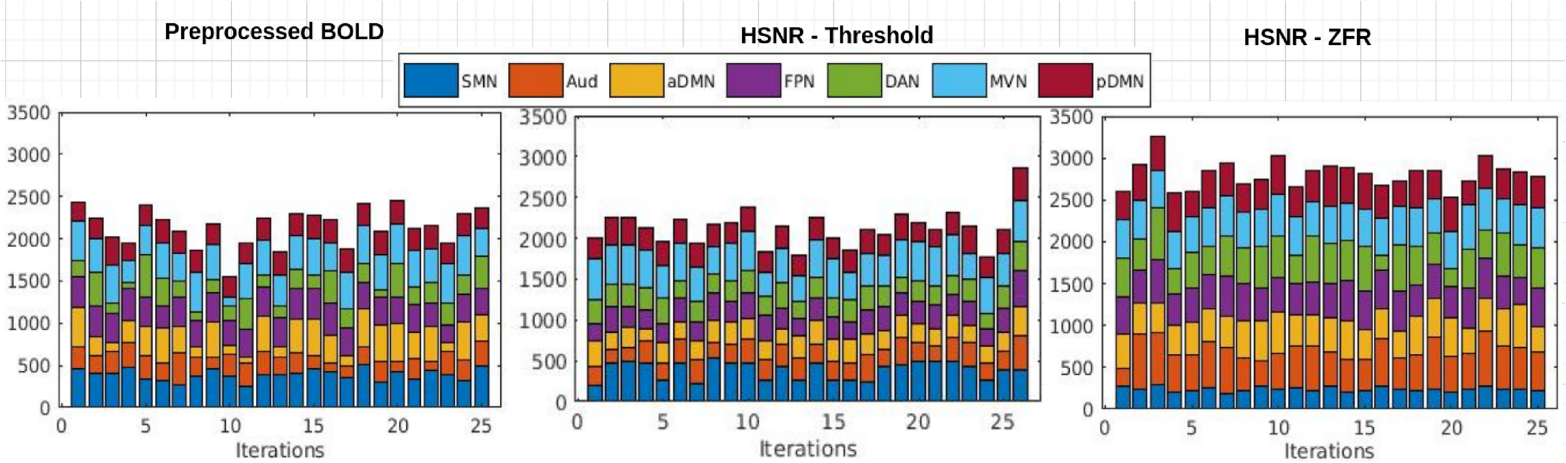
Dwell time (in terms of time frames) of different CAPs obtained from different random samples (HCP data set) in 25 iterations. Each iteration accounts randomly 70% of the total samples for deriving CAPs. Dwell time has been demonstrated for every CAP in different iterations using (a) preprocessed BOLD signal, (b) HSNR segments using the threshold method, and (c) HSNR segments using the ZFR‐based method. BOLD, blood oxygen level‐dependent; CAP, coactivation pattern; HSNR, high signal‐to‐noise ratio.

Stability of the caps using the HCP data set has also been illustrated in Figure 12 using random sampling (70% of 200×50=10,000 time frames) in 25 iterations and computing the Jaccard similarity distances between the CAPs for a particular network. It can be noticed that MVN attains the highest similarity (consistent) through the iterations using the ZFR‐based HSNR segments. The average similarities of all the CAPs are 0.55±0.19, 0.51±0.20, and 0.63±0.16 using three representations (preprocessed BOLD, threshold, and ZFR‐based HSNR segments), respectively. It can be observed that the mean similarity is high and variation is small for ZFR‐based HSNR representation and the same was noticed in the case of the UCLA data set. To quantify the brain dynamics, dwell time of the CAPs has also been illustrated in Figures 13 and 14 for UCLA and HCP data sets, respectively. The seven dominant resting‐state CAPs are illustrated in the figures. It can be noticed that the number of time frames engaged in forming the dominating CAPs is smaller in the case of preprocessed BOLD than in the other two. Using the three representations, the time fraction is more for DMN (including both the aDMN and pDMN). The visual network also persists for a long time in the case of preprocessed BOLD, but Aud network extends more than the preprocessed BOLD for the HSNR cases. The percentage of the fractional occupancy (PFO) for a CAP c is computed as $\mathrm{PF}O_{c}=\frac{100}{Nn_{\mathrm{it}}}\sum_{i=1}^{n_{\mathrm{it}}}n_{c}\left(i\right),$ where, $N\left(=5960\right)$ is the total number of time frames (for UCLA data set), $n_{c}\left(i\right)$ is the number of time frames in the cluster c at *i*th iteration and $n_{\mathrm{it}}$ is the number of iterations. The PFOs are (SMN =2.54, Aud =3.60, aDMN =2.38, FPN =2.14, DAN =2.25, SVN =5.44, and pDMN =3.72), (SMN =4.05, Aud =6.83, aDMN =3.11, FPN =4.24, DAN =3.48, SVN =6.58, and pDMN =5.06), and (SMN =3.66, Aud =6.85, aDMN =2.77, FPN =3.87, DAN =3.52, SVN =6.47, and pDMN =4.72) for the three different representations respectively using UCLA data set. It can also be observed that variations of the dwell time (dwell time for CAP c and at iteration i is the number of time frames out of N time frames falls in the cluster c at *i*th iteration) are smaller in the case of ZFR‐based HSNR representation.

Using the three representations, the time fraction is more for DMN (including both the aDMN and pDMN) for HCP data set. The visual network (MVN) also persists for a long time in all cases. Percentage of the fractional occupancies are (SMN =0.27, Au =0.15, aDMN =0.21, FPN =0.22, DAN =0.16, MVN =0.27, and pDMN =0.18), (SMN =0.27, Au =0.17, aDMN =0.17, FPN =0.18, DAN =0.17, MVN =0.29, and pDMN=0.19), and (SMN =0.16, A u=0.32, aDMN =0.27, FPN =0.29, DAN =0.29, MVN =0.31, and pDMN =0.26) for the three different representations, respectively.

## 4 DISCUSSION

PPA is crucial in rs‐fMRI analysis because of the significant amount of data reduction, increasing the signal‐to‐noise ratio, and getting a neuronal surrogate that can be used in blind deconvolution to estimate NAS. It can reduce more than 50% of the data and produce reliable RSNs without any significant loss of activity information. These values were obtained as a percentage of the average number of the considered time points, that is, the estimated onsets or HSNR time points in the fMRI time courses. Since it can capture the BOLD events associated with high fluctuations, it is able to increase the SNR ratio by mitigating irrelevant noises. The simple threshold‐based PPA may include redundant points or exclude comparatively low‐valued true points. On the other hand, the ZFR‐based approach can extract the precise temporal onsets of the BOLD events. The estimated temporal events can serve as a neuronal surrogate being prior information regarding the neuronal activity in uncoupling the NAS from the observed rs‐fMRI time course. In rs‐fMRI, there are no external stimuli exposed in the experimental paradigm. Therefore, the estimation of reliable onsets of the BOLD (temporal) events is essential to derive the FC itself and deconvolving the underlying NAS whereas a threshold‐based PPA can lead to false negatives.

In the simulated data, connectivity scores using ZFR‐based HSNR correlation show less variation inside the networks, and the ZFR‐based CR shows the highest mean and smaller variance compared to threshold‐based CR (Figure 4). For real rs‐fMRI data, we can observe that the ZFR‐based CR reveals the highest difference between the mean of coactivation and non‐coactivation scores (can be seen in Table 1). The HSNR correlation using less number of time points has also shown a good separation between the means of coactivation and non‐coactivation scores in comparison with correlation scores obtained with the entire time course. This observation has been noticed across the seven dominant resting state networks. The atlas based‐seven networks show comparatively similar or better Fisher discriminant ratio to distinguish between coactivation and non‐coactivation scores using ZFR‐based metrics with a subset of time points.

The extracted events can derive the static as well as dynamic FCs using real rs‐fMRI data. The ZFR‐based CR can distinguish the coactivation and non‐coactivation scores well (can be seen in Figure 2). A few dominant static RSNs demonstrated in Figures 5 and 6 imply that the proposed method can estimate FCs with fewer time points. Dynamic characteristics of the rs‐fMRI in terms of CAPs use the HSNR temporal segments and it reveals FCs by mitigating spurious contents (SMN, aDMN, FPN, SVN, and pDMN in Figures 8 and 9).

ZFR‐based HSNR segments derived stable CAPs (can be seen in Figures 11 and 12). All representations (preprocessed BOLD, threshold, and ZFR‐based HSNR segments) show that the most stable CAP corresponds to the SVN. The least stable CAP is DAN obtained using preprocessed BOLD, whereas the SMN shows the least stability using both HSNR representations. The HSNR representation, derived using ZFR‐based approach, showed the highest mean similarity (0.81) throughout the CAPs. The dwell times in Figures 13 and 14 reveal that temporal noises (possibly not related to the neuronal component) in the preprocessed BOLD contribute to the frames associated with the non‐dominant CAPs in the resting state. In contrast, the HSNR representations include more frames in constituting the dominant resting‐state CAPs. Overall, the dominant CAPs obtained using the ZFR‐based HSNR representation more consistently contribute to time frames throughout the iterations.

It was observed that the average number of extracted onsets for the time course is 12 and 25 using the ZFR and thresholding approaches, respectively. Therefore, the CR (ZFR), CR (Threshold), HSNR correlation, and correlation with the entire time course use ~8%, ~17%, ~44.6%, and ~100% of the entire BOLD signal to derive the connectivity strength respectively (These values were obtained as a percentage of the average number of the considered time points, that is, the estimated onsets or HSNR time points in the fMRI time courses). Although there was a significant reduction in the number of data points used, overall no significant differences in the connectivity maps were observed among the last two cases. With the increasing use of high‐speed imaging with a great number of time points, ZFR methods may provide an efficient analysis pipeline.

The proposed method performs PPA activity precisely. Low temporal resolution rs‐fMRI data may affect the event estimation using ZFR, but the data used in the current study were from a 3 T scanner which is widely used in most fMRI studies. With higher field or spatial/temporal resolution, fMRI data can further enrich the event extraction from rs‐fMRI. The estimation of the spontaneous onsets of the BOLD events uses a window at the output of the ZFR for local mean subtraction which performs a differential operation in the time domain (Yegnanarayana & Murty, 2009). In determining the length in the study, if we choose a small window for ZFR, it may result in spurious onsets (False positives). On the other hand, if we take a long window, it may miss the transient locations in the BOLD response (False negatives). It has been experienced while experimenting with the task fMRI and comparing the estimated onsets of the BOLD events with the stimulus timings (Das et al., 2023). We have empirically varied the window length ($w_{z}$) around the length which we can get from the idea of average samples in the BOLD time course for an event to occur and choose the optimal size of the window (nine samples) for both the synthetic and experimental rs‐fMRI data. The length of the HSNR region is also chosen empirically. It has been chosen based on the following observations: the BOLD time course is modeled as the convolution of HRF and neuronal activity signal, a temporal event of excitation may attain high values as long as the length of the full width of half max (FWHM) of the HRF. The maximum FWHM can be up to 12 s and for TR = 2, it can take a maximum of six samples. In this work, we kept the length of HSNR segment as 6 TR for rs‐fMRI based on the FWHM and a reliability test (given in the supplementary section). With a shorter TR, there will be less sampling error, and the correlation leading to more reliable estimates. However, shorter TR may lead to low SNR and may also introduce additional noise sources including cardiac and respiration signals. As the data becomes available, we may have to modify it to optimize the performance. While we extracted the onsets of the BOLD event in the rs‐fMRI, it does not necessarily prove that they are neurophysiological in nature. For future studies, experimental studies could be designed that will be able to control/modulate the firing rate in the resting state signal. The proposed work does not address the spatiotemporal extent of neural activity. The formulation of the fMRI time‐series model given by Equation (1) with the hypothesis that the NAS or activity‐inducing signal has the characteristics of impulses that get sluggish by the hemodynamic activity. It is agreed that the rs‐fMRI has temporal dependence and these temporal dependencies contribute to FC. Consequently, removing discrete time points will not significantly affect the connectivity between time series as they are indeed an output between neuronal events convolved with a sluggish hemodynamic function. However, it must be emphasized that this work has focused on the time‐series model, from which we have tried to extract information regarding the NAS in rs‐fMRI. In recent years, several methods describing the spatiotemporal structure of neural activity have been presented in the literature. Recently, Pang et al. (2023) used the geometric shape of the cortex to develop the spatiotemporal model of resting‐state connectivity. Similarly, Raut et al. (2021) and Bolt et al. (2022) have empirically shown that resting‐state fMRI signal fluctuations can be accounted for by large‐scale waves of activity propagating throughout the brain. All these methods including ours have made some assumptions regarding the origins of these fluctuations. While we have used a mathematical‐based model where each time series is used to infer about the NAS, there have been mechanistic models that have been developed that use noise signals to generate connectivity. It is our hope that we can complement these methods to develop better spatiotemporal models of connectivity.

In this work, our main objective is to extract these BOLD events from the rs‐fMRI time course. Specifically, our hypothesis is that once the spontaneous neuronal activity occurs, it leads to an increased blood flow leading to the transient behavior in the BOLD response (we call it a BOLD event). The work reported in this manuscript is about the extraction of BOLD events from the rs‐fMRI time course, while the work of Zamani Esfahlani et al. (2020) and Ladwig et al. (2022), demonstrate the contribution of high/low or in between high and low co‐fluctuation values in edge time series to derive FC. A transient activity in the time course (called a BOLD event) will have immediate dependencies with the predecessor or successive BOLD signal responses. This phenomenon makes the non‐event time points that are good in numbers in an rs‐fMRI time course for a particular voxel/ROI and leads to significant connectivity values between voxels/ROIs. Therefore, FC can still be reliably estimated using BOLD values after removing the high fluctuation values (Ladwig et al., 2022). However, these values could be sensitive to noise and may give spurious FC in the presence of noise.

The onsets of the spontaneous BOLD events in the rs‐fMRI can be precisely estimated using the ZFR‐based approach. The extracted events and the temporal HSNR regions around the onsets can derive reliable static (CRM) and dynamic (CAP) FC. The estimated static FC was able to differentiate between coactivation and non‐coactivation in the distribution of CR. The HSNR‐driven CAPs also demonstrate higher consistency and time occupancy by the dominant resting state networks.

For resting state fMRI, the ratio of mean percentage signal change within HSNR regions to the mean percentage signal change in non‐HSNR regions could be a potential measure of the data quality especially when noise is higher. As the BOLD activity changes across different pathological cases, the difference between the mean BOLD responses in the HSNR region and the non‐HSNR regions can be used as an rs‐fMRI‐based marker in diagnosing pathological cases.

The work can also be extended in estimating parametric HRF using ZFR‐driven BOLD events. The ZFR extracts the transient locations in the fMRI time course and is called the estimated BOLD events. The sequence of the events driven by the ZFR can be used to construct meaningful regressors by convolving it with the HRF basis (for parametric HRF) in the GLM which yields a precise estimate of the HRF. The sequence of the onsets of the BOLD events can serve as a promising neuronal surrogate in deconvolution. Potential studies have been carried out to estimate the NAS using an artificial neuronal activity surrogate (Wu et al., 2013). In deconvolution, the approach has to employ a suitable neuronal surrogate which is obtained by fixing a predefined threshold. While considering the high‐valued points using thresholding are justified, it may raise false positives in the neuronal surrogates (Das et al., 2023). Instead of the threshold‐based neuronal surrogate, the ZFR‐based neuronal surrogate can be used as prior information in the deconvolution of the NAS. The sequence of the estimated onsets of the BOLD events can also be employed to increase the efficacy in estimating the NAS using nonparametric deconvolution introduced in the work (Das et al., 2021). There, the estimated sequence using the ZFR can play an important role in determining the cutoff quefrency to separate the NAS and the HRF in the cepstrum domain.

## AUTHOR CONTRIBUTIONS

**Sukesh Kumar Das:** Conceptualization of this study; methodology; implementation; original draft preparation. **Anil K. Sao:** Conceptualization; draft review and editing. **Bharat B. Biswal:** Investigation; draft review and editing.

## CONFLICT OF INTEREST STATEMENT

The authors declare no conflicts of interest.

## Supporting information


**Data S1.** Supporting Information.

## Data Availability

The data that support the findings of this study are openly available in UCLA consortium for Neuropsychiatric phenomics data set at https://openneuro.org/datasets/ds000030/versions/00016.

## Footnotes

^1^

http://www.fil.ion.ucl.ac.uk/spm/.
