# Supplementary material for: Estimation of static and dynamic functional connectivity in resting‐state fMRI using zero‐frequency resonator
Source: Hum Brain Mapp. 2024 Jun 19;45(9):e26606. doi: 10.1002/hbm.26606 (PMC11187872; doi:10.1002/hbm.26606)
Supplement: Supplementary file 1 — Data S1. Supporting Information. [file HBM-45-e26606-s001.pdf]

Supplemental Material

Estimation of static and dynamic functional connectivity in resting state fMRI using zero frequency resonator

Sukesh Kumar Das, Anil K. Sao, Bharat Biswal

1.1 Why use a ZFR?

Our work is built on the hypothesis (Eq. 1 of the manuscript) that the neuronal activity signal (NAS) or activity-inducing signal (AIS) has the characteristic of impulses and it is reflected at the transient location in the BOLD response with a small delay as the BOLD contrast is the consequence of the local spontaneous-neuronal activity. A resonator produces a single frequency magnitude peak at a given specific frequency. Now, An ideal impulse has an equal energy at all frequencies including around the zero frequency in the spectrum. So, the output of a zero-frequency resonator (ZFR) provides the information of onsets of the transient behavior in the BOLD response at its output. Thus ZFR can be used to estimate the instantaneous changes (events) in the time course.

1.2 How was it derived?

The ZFR is a second-order IIR (infinite impulse response) filter having a complex conjugate pair of poles located inside the unit circle of the z-plane. The angle (ω in Fig. S1) of the poles with the abscissa of the z-plane decides the resonant frequency of the resonator, while the distance (r in Fig.S1) of the poles in the unit circle sets the bandwidth. The closer it is to the unit circle, the smaller the bandwidth. The transfer function of a resonator having a pair of complex conjugate poles at r cos(ω) ± jr sin (ω) is

$$H_r(z) = \frac{1}{\left(1-(rcos(\omega)+jrsin(\omega))z^{-1}\right)\left(1-(rcos(\omega)-jrsin(\omega))z^{-1}\right)} = \frac{1}{1-2rcos(\omega)z^{-1}+r^2z^{-2}}$$

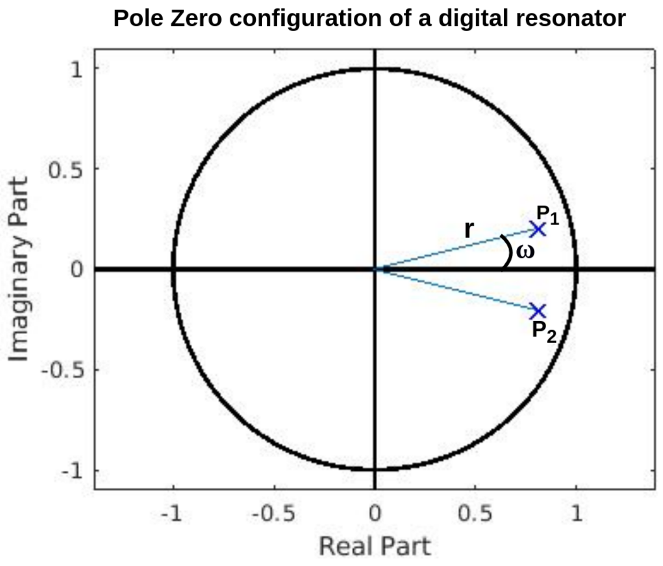

Figure S1: Pole-zero configuration for a digital resonator whose resonant frequency is ω

For an ideal resonator (minimum width) with zero resonant frequency, the value of r and ω would be one and zero respectively. Thus transfer function of the ZFR is

$$H_z(z) = \frac{Y(Z)}{M(Z)} = \frac{1}{1-2z^{-1}+z^{-2}}$$

where Y (z) and M (z) are the z-transform of output y[n] and input m[n] of a ZFR respectively.
In the time domain

$$y[n] = m[n] + 2y[n-1] - y[n-2]$$

The recurrence relation can be interpreted as the cumulative sum of the input performed twice. Thus, the output of ZFR for a BOLD time course will grow approximately as a polynomial function of time. But the fluctuations in the output contain the information of BOLD events (temporal). In order to extract the information, the local mean is subtracted from the output signal [Yegnanarayana and Murty, IEEE Trans Audio, Speech, and Language, 2009] and we call it zero frequency filtered signal (ZFFS), $z[n] = y[n] - \frac{1}{2N_1+1}\sum_{k=-N_1}^{N_1} y[n-k]$, where $(w_z = 2N_1 + 1)$ is the size of the window in samples.

Zero crossing points in the ZFFS provide the onsets of the BOLD events. We have illustrated the outputs of the different steps of the ZFR in Fig. S2 for a resting state BOLD time course.

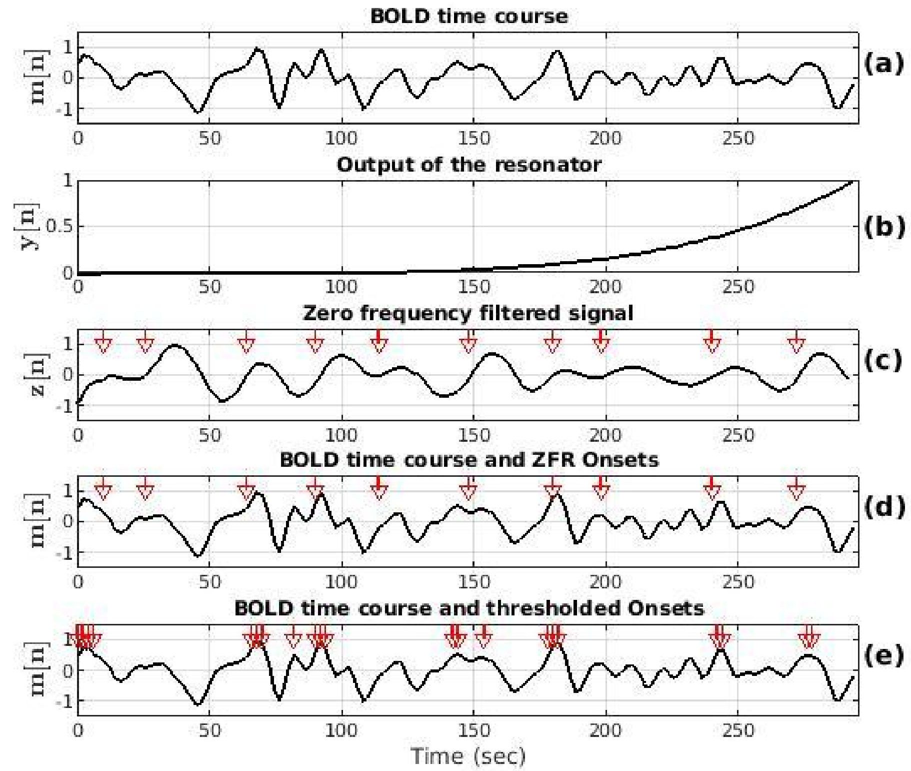

Figure S2: Output of the steps involving the ZFR for a resting state time course taken from the right lateral parietal cortex (rLPC): (a) Resting state BOLD time course, (b) output of the resonators (growing), (c) Zero Frequency Filtered signal: ZFR output after local mean subtraction and estimated onsets (red markers) at positive zero crossing. The window length for the rs-fMRI data is $w_z = 9$, (d) Resting-state BOLD time course and location of the onset (red markers) estimated by ZFR, and (e) Resting state BOLD time course and location of the onset (red markers) estimated by the thresholding (red markers)

2. Length of the window for the HSNR segment:

Deciding a suitable sample size, for HSNR correlation, depends on a number of factors e.g. exact population size, the purpose of study, and precision or confidence interval. In this study, it is not known what is the maximum HSNR sample per neuronal event (temporal). So, population size is hard to determine but as the BOLD time course is modeled as the convolution of HRF and neuronal activity signal, a temporal event of excitation may attain high values as long as the length of the full width of half max (FWHM) of the HRF. The maximum FWHM can be up to 12 sec and for TR=2, it can take a maximum of 6 samples (we can consider it as the population size).

For the reliability test, we considered 70% of the data (event-related finger tapping task described in Das. et. al, NeuroImage, 2023) using random sampling and got 10 combinations in 10 iterations for each of which we estimated the average HSNR correlation map (motor network). These 10 connectivity maps yield 45 (=10*(10-1)/2) Jaccard similarity distances (JSD). For different lengths of high SNR samples, we have computed the distances (between maps) as shown in **Fig. S3** below. We have also shown the variances using the different lengths of the HSNR segment in the below table. It shows that length 6 (-1 0 1 2 3 4) shows comparatively less variance in JSD along the different combinations of iterations. 0th location indicates the location of the estimated onsets. Here, it can be observed that the onset point is not in the center of the window. The reason behind it is that the ZFR estimates the onsets mostly at the transient location of the BOLD response (high transition point at the rising slope).

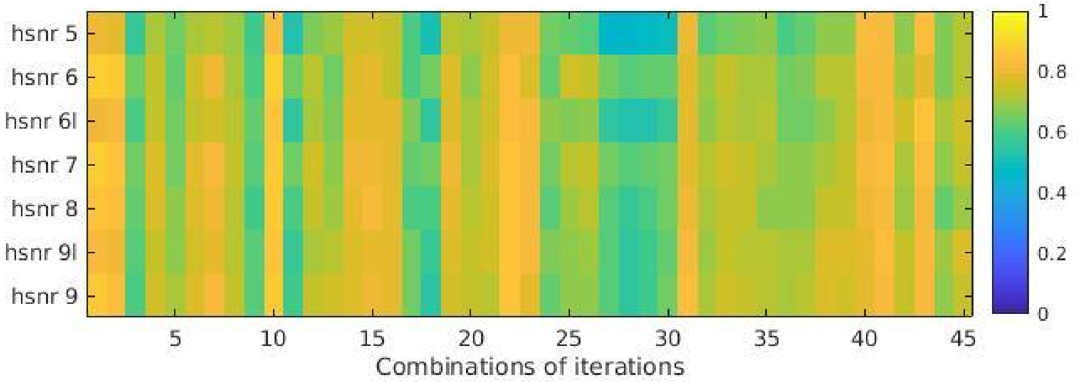

Figure S3: JSD between the connectivity maps. 10 iterations give 45 distances for each HSNR length.

Table: Variation of JSDs

**Length (samples location around onsets, estimated onset location is on 0)**	**Variance**
hsnr 5 (-1 0 1 2 3)	0.102
hsnr 6 (-1 0 1 2 3 4)	0.075
hsnr 6l (-2 -1 0 1 2 3)	0.088
hsnr 7 (-2 -1 0 1 2 3 4)	0.076

hsnr 8 (-2 -1 0 1 2 3 4 5)	0.083
hsnr 9l (-3 -2 -1 0 1 2 3 4 5)	0.078
hsnr 9 (-2 -1 0 1 2 3 4 5 6)	0.083

3. The optimal number of clusters using Elbow method:

For estimating the optimal number of clusters, distortions were computed for different values of k(= 15, 16, ., ., ., 29, 30). The value of K at the lowest distortion was 28 (Fig. S4) and was considered the optimal number of clusters.

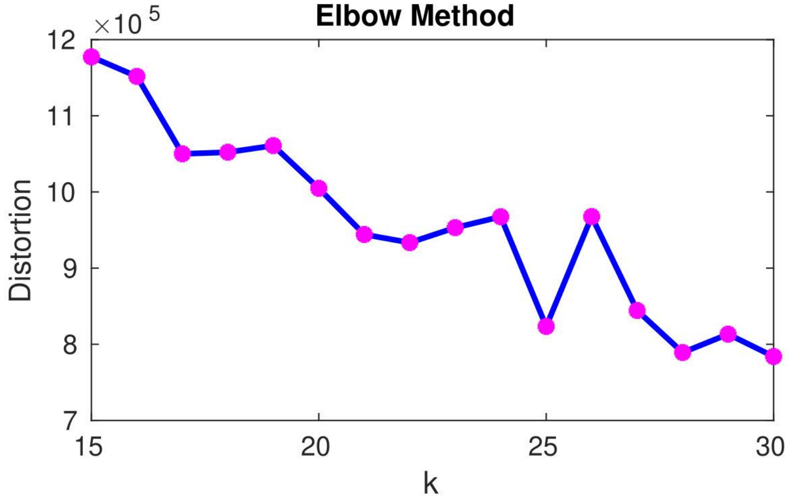

Figure S4: Elbow curve: Distortion for different values of k.

Using the optimal number of clusters (i.e. 28) the CAP analysis was performed in the manuscript. Obtaining the optimal number of k using the elbow method has been illustrated in the supplementary section.

4 Experiment on HCP Dataset:

We have performed all the analyses using HCP resting state data. We have also included it in the main manuscript. As it is here earlier, we are not removing it from here.

4.1 Data Description:

We assessed the 50 unrelated subjects (25 females, 25 males, mean age = 29.1 ± 3.7 years) from the HCP 900 subjects *dataset*[1] released. The fMRI resting-state run (HCP filename: rfMRI_REST1) was used, with the average of two different acquisitions (left to right or LR and right to left or RL) of 200-time points. Subjects were asked to keep their eyes open with a fixation on a white cross. The fMRI data were acquired using the following scan parameters: Repetition time (TR)=720 ms, Slice thickness = 2.0 mm, TE (echo time) = 33.1 ms; field of view = 208x180 mm (RO x PE); flip angle = 52 ∘ ; # slices (axial) = 34; slice thickness = 2 mm; matrix size = 104 × 109 (RO x PE); #images = 1200 (In this study 200 images are used). The static and dynamic FCs are demonstrated in the following Figs. S5 - 6.

1. (https://www.humanconnectome.org/storage/app/media/documentation/s900/HCP_S900_Release_Reference_Manual.pdf)

4.2 Static functional connectivity:

Seven resting state FCs (central executive network (CEN), medial sensory-motor network (MSMN), auditory network (Aud), default mode network (DMN), frontoparietal network (FPN), dorsal attention network (DAN) and superior visual network (SVN)) are demonstrated in Fig. S5. It can be noticed that the corresponding FCs can be derived using a few inflection points obtained by ZFR.

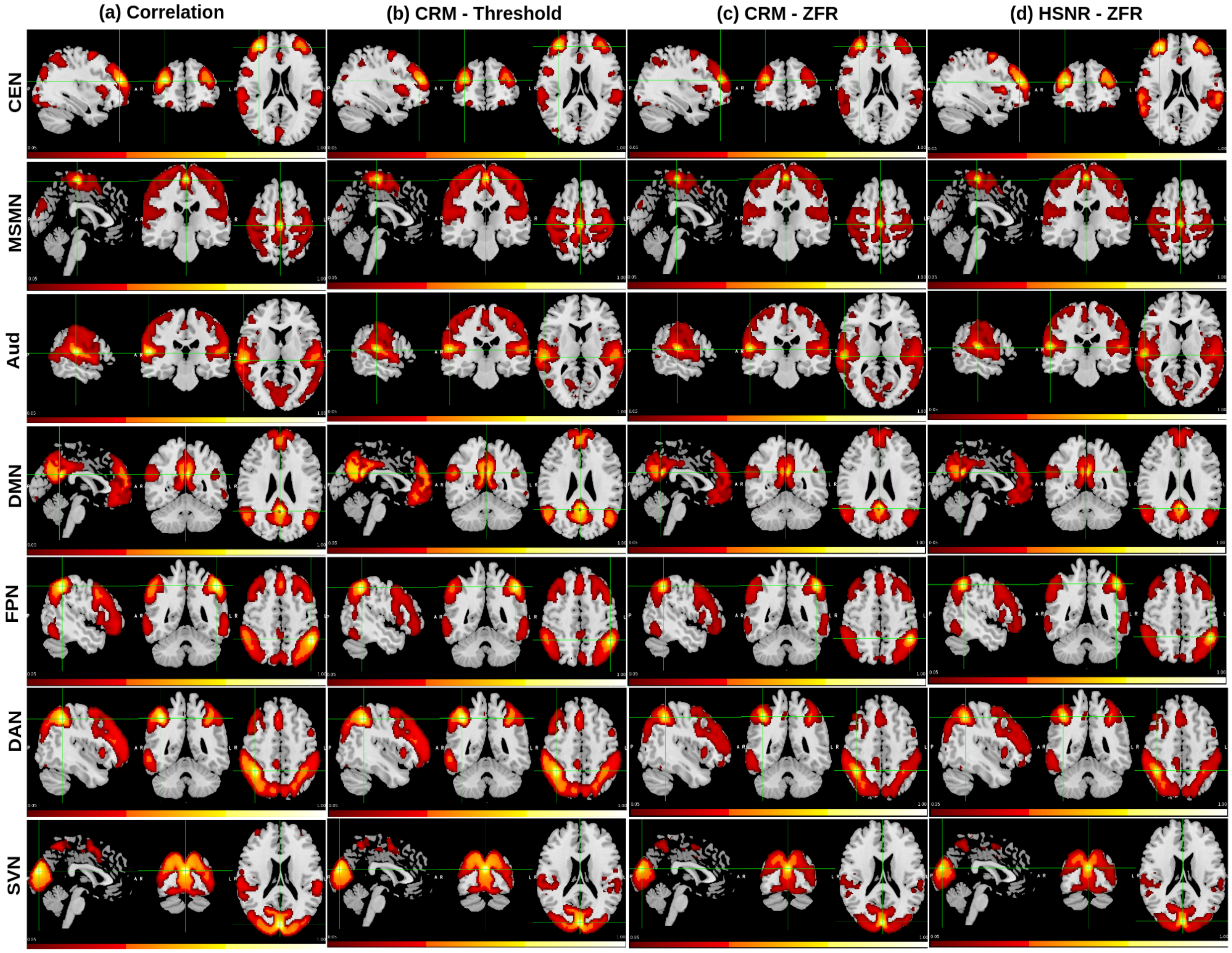

Figure S5: Static FCs obtained using the 50 subjects' data taken from HCP dataset. Sagittal, coronal and axial views of the FC with respect to seven seeds (from top: CEN - MNI co-ordinate 34, 46, 20, MSMN - MNI co-ordinate 0, −24, 60, Aud - MNI co-ordinate 58, −24, 11, DMN - MNI co-ordinate 0, −52, 26, FPN - MNI co-ordinate −48, −47, 49, DAN - MNI co-ordinate 40, −47, 48 and SVN - MNI co-ordinate 0, −85, 20). (a) Correlation map using entire time courses, (b) and (c) CRMs using the estimated onsets by threshold and ZFR respectively and (d) Connectivity map using an average of aggregated correlation in HSNR segments estimated by ZFR. All connectivity maps are presented using the same scale (0.05 − 1).

4.3 Dynamic functional connectivity (CAP):

CAPs are derived from a total of 10000 (200x50) time frames using the three representations (from left to right: Preprocessed BOLD, threshold, and ZFR-based HSNR segments respectively). Identified CAPs corresponding to

different dominating resting state networks are obtained using a subset of time points estimated by the ZFR and are demonstrated in Fig. S6. From top to bottom the CAPs are sensory motor network (SMN), auditory network (Aud), anterior default mode network (aDMN), frontoparietal network (FPN), dorsal attention network (DAN), medial visual network (MVN) and posterior DMN (pDMN).

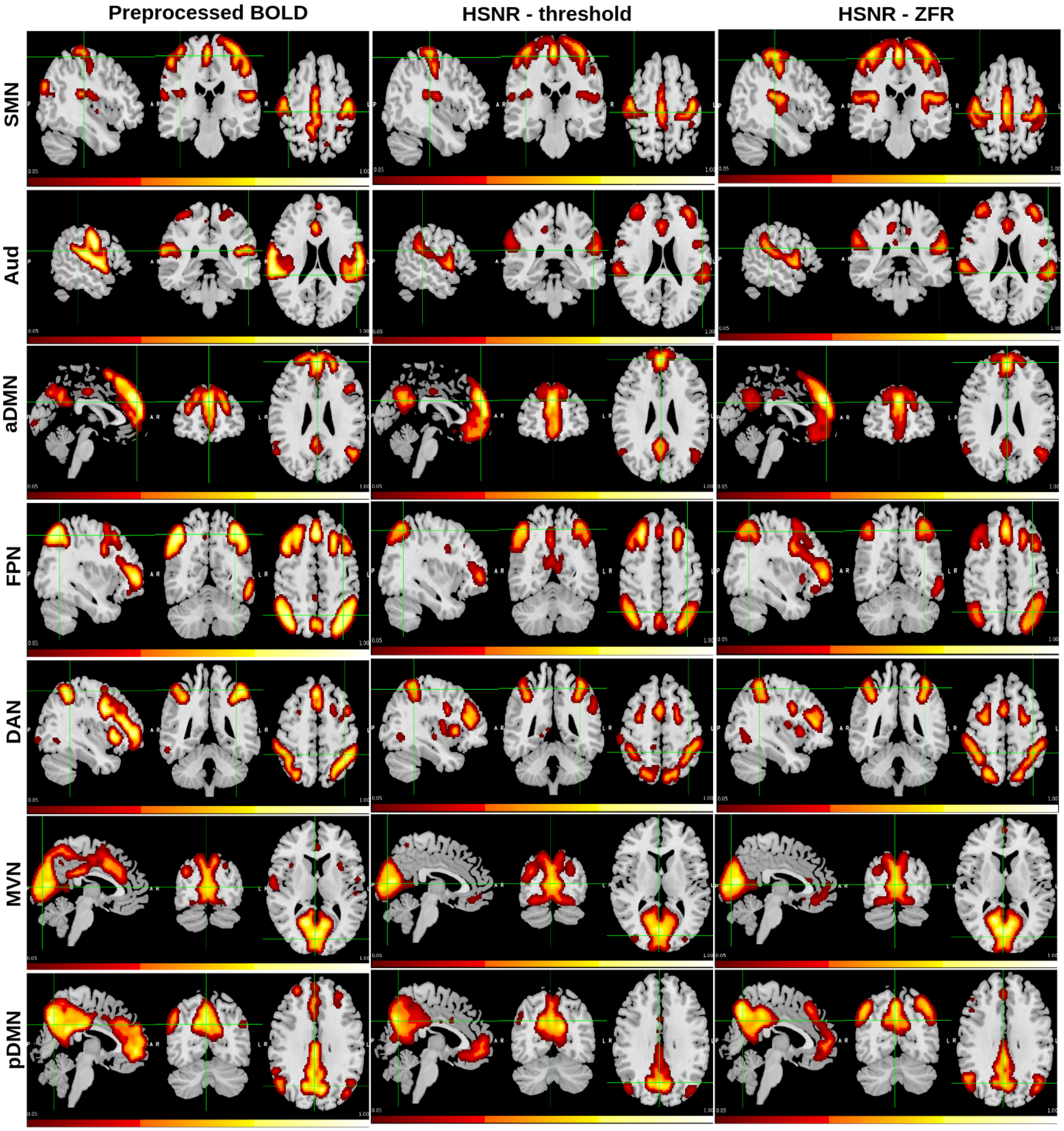

Figure S6: CAPs obtained using the 50 subjects' data taken from HCP dataset. Sagittal, coronal and axial views of the CAPs: Seven rows correspond to seven CAPs (from top SMN, Aud, aDMN, FPN, DAN, MVN (medial visual network) and pDMN). CAPs are obtained using (a) preprocessed data, (b)HSNR segments around the onsets estimated by the thresholding and (c) HSNR segments around the onsets estimated by the ZFR in the rs-fMRI time course

4.4 Stability of the CAPs:

Stability of the caps using the HCP dataset has been illustrated in Fig. S7 using random sampling (70% of 200x50=10000 time frames) in 25 iterations and computing the Jaccard similarity distances between the CAPs for a particular network. A total of $(n_{it}(n_{it}\text{-}1)/2 = 300)$ similarity distances were obtained for a network in n_{it}=25 iterations and shown in Fig. S7. It can be noticed that MVN attains the highest similarity (consistent) through the iterations using the ZFR-based HSNR segments. The average similarities of all the CAPs are 0.55±0.19, 0.51±0.20 and 0.63±0.16 using three representations (Preprocessed BOLD, threshold, and ZFR-based HSNR segments) respectively. It can be observed that the mean similarity is high and variation is small for ZFR-based HSNR representation and the same was noticed in the case of the UCLA dataset.

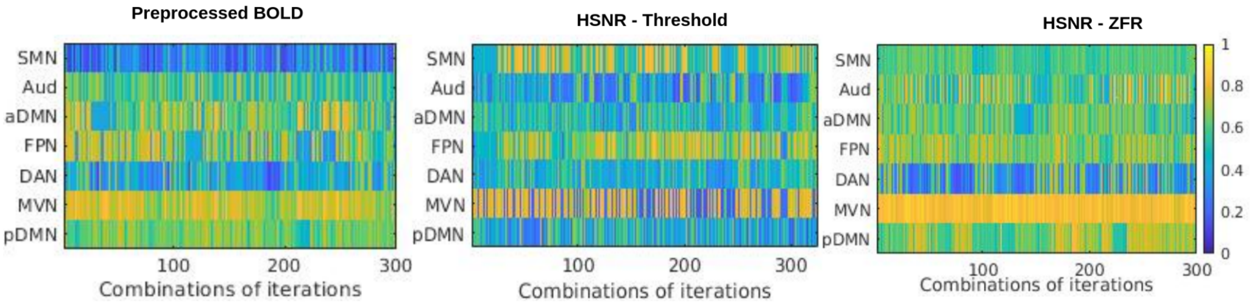

Figure S7: Jaccard similarity distances between CAPs obtained from different random samples (HCP data). Total 25 iterations and randomly 70% of the total samples were considered for deriving CAPs in every iteration. A total of 300 similarity values have been demonstrated for every CAP. CAPs are derived using (a) preprocessed BOLD signal, (b) HSNR segments using the threshold method, and (c) HSNR segments using the ZFR-based method.

Dwell time of the CAPs from HCP data has also been illustrated in Fig. S8. Using the three representations, the time fraction is more for DMN (including both the aDMN and pDMN). The visual network (MVN) also persists for a long time in all cases. Percentage of the fractional occupancies are (SMN=0.27%, Au=0.15%, aDMN=0.21%, FPN=0.22%, DAN=0.16%, MVN=0.27%, pDMN=0.18%), (SMN=0.27%, Au=0.17%, aDMN=0.17%, FPN=0.18%, DAN=0.17%, MVN=0.29%, pDMN=0.19%), and (SMN=0.16%, Au=0.32%, aDMN=0.27%, FPN=0.29%, DAN=0.29%, MVN=0.31%, pDMN=0.26%) for the three different representations respectively.

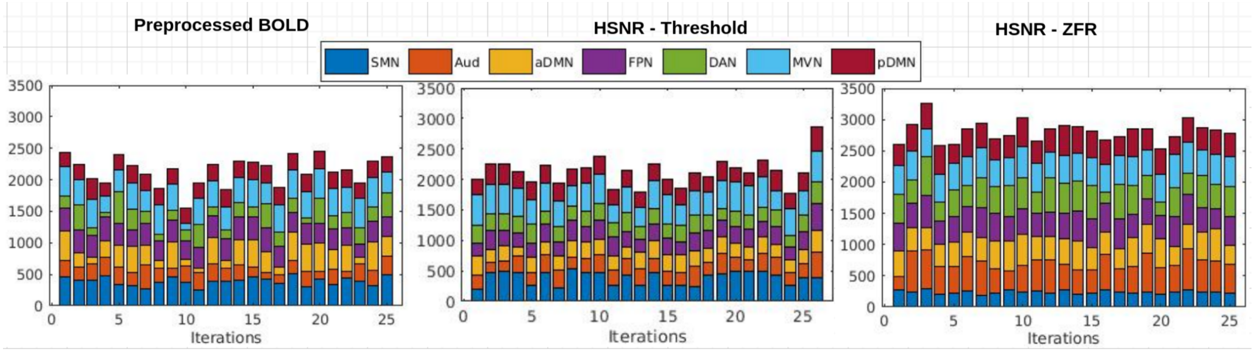

Figure S8: Dwell time (in terms of time frames) of different CAPs obtained from different random samples in 25 iterations (on HCP data). Each iteration accounts randomly for 70% of the total samples for deriving CAPs. Dwell time has been demonstrated for every CAP in different iterations using (a) preprocessed BOLD signal, (b) HSNR segments using the threshold method, and (c) HSNR segments using the ZFR-based method.

5. Generation of the synthetic data:

The synthetic fMRI time courses (TCs) are generated using the SimTB, which follows an inverse procedure of the ICA (independent component analysis) [Erhardt, Allen, Wei, Eichele and Calhoun (NeuroImage 2012)]. Here spatial components are selected and intersubject variability is introduced to the spatial components by spatial variability in translation, rotation, and spread. Then event time series (TS) are generated and convolved with the hemodynamic response function (HRF) model to get component time courses (TC). Voxelwise time courses are then generated as a linear combination of spatial components weighted by the component-time courses with added baseline tissue weights. Finally, Rician noise was added to the simulated data with random contrast-to-noise ratios.

As the resting state-fMRI is assumed as event-related, we have followed the event-related experimental design to form TS. In the event-related paradigm, four types of stimuli are presented to each subject in random order: the standard stimulus, target stimulus, novel stimulus and spike event. The standard stimulus is a baseline tone that occurs frequently. The target stimulus is a distinct tone that the subject should press a button upon hearing and the novel stimulus is a random digital noise. The tool models distinct effects of standard, target, and novel tones on the BOLD signals of different sources. The spike event occurs rarely and is assigned only to CSF source (component) to match the time-course skewness of CSF seen in real data.

The probabilities of the four events are 0.6, 0.075, 0.075, and 0.05 respectively. These probabilities represent the chances of occurring the events at each TR. The event probability and event amplitude are used to produce the event TS. For each component, generating the fMRI BOLD-like time courses (TCs) from the event TS may be done by linear convolution with a canonical HRF. Standard events are mapped to auditory sources (rAud and lAud) with amplitude 1 and DMN, with amplitude -0.3, . Target events are mapped to auditory sources (rAud and lAud) with amplitude 1.2, motor sources (M1 and M2) with amplitude 1, and other sources (DMN, DAN) have amplitude -0.3 and 0.8 respectively. Novel events are mapped to auditory sources (rAud and lAud) with an amplitude 1.5, motor sources (M1 and M2) with an amplitude 0.5, and other sources (DMN and DAN) have an amplitude -0.3 and 1.2 respectively. The DMN source is negatively activated with all three sources having amplitude −0.3. Spike events are mapped only to the CSF sources (CSF1 and 2) with amplitude 1. A sample synthetically generated time course has been demonstrated in Fig. S9.

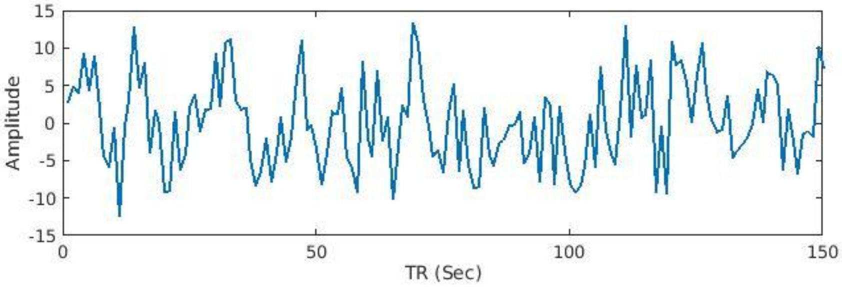

Figure S9: Synthetic time course generated using the SimTB. The time course is taken from the left auditory component
